# Ferroptosis-Related lncRNAs Act as Novel Prognostic Biomarkers in the Gastric Adenocarcinoma Microenvironment, Immunotherapy, and Chemotherapy

**DOI:** 10.1155/2023/9598783

**Published:** 2023-05-19

**Authors:** Yushi Zheng, Shanshan Wu, Xueshan Huang, Lianxiang Luo

**Affiliations:** ^1^The First Clinical College, Guangdong Medical University, Zhanjiang, Guangdong 524023, China; ^2^Department of Biology, School of Basic Medical Science, Guangdong Medical University, Zhanjiang, Guangdong 524023, China; ^3^The Marine Biomedical Research Institute, Guangdong Medical University, Zhanjiang, Guangdong 524023, China; ^4^The Marine Biomedical Research Institute of Guangdong Zhanjiang, Zhanjiang, Guangdong 524023, China

## Abstract

Ferroptosis, a form of programmed cell death akin to necrosis, is managed by iron and is distinguished by lipid peroxidation. Gastric cancer is a highly aggressive form of cancer, responsible for the third highest number of cancer-related deaths globally. Despite this, the potential of ferroptosis to predict the occurrence of this cancer is yet to be determined. In this research, a comprehensive examination was conducted to explore the link between long noncoding RNAs (lncRNAs) and ferroptosis, in order to uncover an lncRNA signature that can predict drug susceptibility and tumor mutational burden (TMB) in gastric adenocarcinoma. We conducted an in-depth analysis of the GC immune microenvironment and immunotherapy, with a particular focus on ferroptosis-related lncRNA prognostic biomarkers, and further explored the correlation between these factors and prognosis, immune infiltration, single nucleotide variation (SNV), and drug sensitivity for gastric adenocarcinoma patients. Through our investigations, we have discovered five lncRNA signatures related to ferroptosis that can accurately forecast the prognosis of gastric adenocarcinoma patients and also regulate the proliferation, migration, and occurrence of ferroptosis in gastric adenocarcinoma cells. In conclusion, this lncRNA signature associated with ferroptosis may be employed as a prognostic indicator for gastric adenocarcinoma, thus presenting a potential solution.

## 1. Introduction

Gastric cancer (GC) is one of the five most commonly diagnosed diseases and the third leading cause of cancer-related fatalities across the globe, making it a major challenge for oncology [1]. Gastric adenocarcinomas (STAD) account for approximately 95% of all GC cases [2]. Most of the early symptoms of cancer are not evident, owing to the fact that the majority of patients are diagnosed at an advanced stage when the prognosis is poor, and the treatment options are limited [3]. Regrettably, the tumor markers most commonly used for the initial clinical diagnosis of GC have a low level of sensitivity and specificity [4, 5]. Consequently, more accurate biomarkers are urgently needed to reflect an individual's cancer risk and to develop new therapeutic strategies.

Generally, GC is a multifaceted condition, which involves a variety of genetic mutations, epigenetic modifications, chromosomal translocations, deletions, and amplifications. These can all be contributory to the genesis of the disorder. In contrast to mutations in or abnormal expression of protein-coding genes, epigenetic modifications, such as the overexpression or downregulation of long noncoding RNA (lncRNA), not only play a role in cancer initiation and progression [6]. Simultaneously, they can also display tumor-suppressive or oncogenic effects. Owing to the genome-wide expression patterns of lncRNAs in diverse tissues, they may be used as biomarkers and therapeutic targets for cancer [7]. Uncovering the key lncRNAs participating in GC progression is essential for comprehending the mechanisms at work.

Ferroptosis, a recently identified form of cell death, is caused by a massive lipid peroxidation process that requires iron, leading to damage of the cell membrane [8]. Ferroptosis, initially induced by the small molecule erastin, is primarily defined by the decrease of a cell's volume and the intensification of mitochondrial membrane density, without the usual signs of apoptosis and necrosis [9]. It has been established in prior research that lncRNAs are connected to a range of PCD phenomena, including apoptosis, autophagy, necroptosis, and ferroptosis. It has been observed that certain lncRNAs can function as competing endogenous RNA, thereby hindering oxidation and thus, ferroptosis; in contrast, some lncRNAs are known to induce autophagy. Research has indicated that examining the correlation between lncRNA and ferroptosis in various cancers, including GC and non-small cell lung cancer, has significant implications [10]. In order to explore the connections between ferroptosis, ferroptosis-related lncRNA, and gastric adenocarcinoma, a comprehensive evaluation is necessary.

The TME consists of several stromal cells that are necessary for cancer cells to flourish and propagate [11]. Accumulating evidence has indicated that gastric adenocarcinoma has a particular microenvironment that facilitates tumor progression and metastasis [12]. It is essential to conduct further research into the connection between the TME and gastric adenocarcinoma, as the exact mechanism of interaction is still unknown. Precision medicine and targeted therapies have been incorporated into medical oncology, leading to a transformation of the way cancer is treated [13]. Precision medicine has exposed significant heterogeneity in cancer pathways gone awry, and the employment of novel targeted therapies, especially immune checkpoint inhibitor therapies whose responsiveness is evaluated using a TIDE score, has been especially effective [14] and has shown even broader prospects in various cancer types [15]. Recently, immune checkpoint inhibitors (ICIs) have been put forward as a possible treatment option for gastric adenocarcinoma. Cytotoxic T lymphocyte-associated antigen-4 (CTLA-4) and programmed cell death protein 1 (PD-1), two immune checkpoints that control lymphocyte activation and balance immune responses, can shield tumor cells from the immune system [16].

Our study makes use of prognostic biomarkers of ferroptosis-related lncRNA to analyze the tumor-immune microenvironment and immunotherapy in a comprehensive manner. Five lncRNA signatures linked to ferroptosis were established, as well as a relevant nomogram. lncRNA signatures associated with ferroptosis have proved to be a reliable predictor of the prognosis of gastric adenocarcinoma patients. We have successfully created a prognostic model to examine the connection between prognosis, immune infiltration, SNV, and drug sensitivity of STAD patients.

## 2. Materials and Methods

### 2.1. Data Acquisition and Preprocess

The Cancer Genome Atlas (TCGA) database (https://portal.gdc.cancer.gov/repository) provided access to the RNA sequencing (HTSeq-Counts), simple nucleotide variation data, and clinical information of 380 TCGA-STAD patients. Afterward, we transformed the count data into FPKM (fragments per kilobase of transcript sequence per millions of base pairs sequenced) in order to carry out the following analysis. The study encompassed 224 samples with comprehensive clinical information (Table 1). The FerrDb database was used to assemble a gene list comprising 291 ferroptosis-related genes (http://www.zhounan.org/ferrdb) [17] and the human gene database (Gene Cards) using the keyword “ferroptosis” (https://www.genecards.org/) [18]. Applying the “edgeR” package, a differential analysis was conducted, which yielded 18 ferroptosis-related differentially expressed genes (DEGs) in TCGA-STAD [19, 20]. False discovery rate (FDR) < 0.05 and |log2 fold change (FC)| ≥ 1 are the statistical parameters for significance. Subsequently, the STRING database (https://string-db.org/) [21] was used to generate a protein-protein interaction network (PPI) of 18 ferroptosis-related genes and ran a Cytoscape plugin, CytoHubba [22], to accurately determine which genes served as hubs in the PPI network. Pearson's correlation analysis (with |Pearson R| > 0.5 and *p* < 0.001) was utilized to identify strong interactions between TCGA-STAD lncRNAs and ferroptosis-related genes, thus allowing the selection of ferroptosis-related lncRNAs. Through differential analysis, we identified 142 upregulated and 121 downregulated ferroptosis-related lncRNAs.

### 2.2. Establishment and Evaluation of a Ferroptosis-Related lncRNA Signature Prognosis Model

Utilizing the criteria stated above, we conducted an analysis of 200 patients with complete clinical information. A total of 200 patients were randomly divided into two groups: a training group of 100 and a test cohort of 100. The clinical characteristics of the training cohort and the test cohort were identical. Through the utilization of univariate and multivariate Cox regression analysis and the “survival” R package, lncRNA signatures pertinent to ferroptosis were determined [23] relevant to the prognosis of gastric adenocarcinoma patients. A prognostic risk model that is based on five ferroptosis-related lncRNAs was developed through multivariate Cox regression analysis, allowing the prediction of the prognosis of individuals suffering from gastric adenocarcinoma. The risk score was obtained by the following equation: Risk score = (exprgene1 × coefficientgene1) + ⋯+(exprgene5 × coefficientgene5). In order to validate the risk characteristic model in the data set, we computed the risk score for each patient with gastric adenocarcinoma in both the training and test sets. By taking into consideration the median value of the risk score, all samples were split into two categories—a high-risk group and a low-risk group—for the purpose of examining the prognosis of individuals suffering from gastric adenocarcinoma. To evaluate the overall survival (OS) of the two patient cohorts, a Kaplan-Meier analysis was performed. We selected the “survminer” R package to calculate the optimal cutoff expression. To evaluate the risk assessment model's independence from other clinical features, a multivariate Cox regression analysis was employed. The AUC analysis evaluated the effectiveness of the ferroptosis-related lncRNA and the validity of this risk model. The Kaplan–Meier analysis was employed to individually assess the five ferroptosis-related lncRNAs, in order to investigate the correlation between their expression and patient survival. Utilizing the “rms” R package, a nomogram was constructed based on risk score and independent clinical information, with calibration curves established for 3, 5, and 7 years.

### 2.3. Comprehensive Immunoassay

A comparison of immune cells between TCGA-STAD groups with high and low risk was conducted with the help of seven algorithms [TIMER [24], CIBERSORT [25, 26], CIBERSORT-ABS, QUANTISEQ [27], MCPCOUNTER, XCELL [28], and EPIC [29]], and the results were visualized by “limma” and “heat map” R package. Applying the “GSVA” R package, single-sample gene set enrichment analysis (ssGSEA) was carried out to assess immune-related cells and pathways in each TCGA-STAD sample [30]. The sample's immune infiltration result was found to be trustworthy, as the *p* value was below 0.05. Samples from the TCGA-STAD were assigned a stromal score, an immunological score, and an ESTIMATE score using the “estimate” R tool [31]. Furthermore, CIBERSORT was employed to illustrate the ratio of 22 different types of immune cells in the sample.

Using the TIDE algorithm (http://tide.dfci.harvard.edu/), to predict the response of each sample in the TCGA-STAD cohort to anti-PD-1 and anti-CTLA4 immunotherapy, we calculated the TIDE scores for each sample. According to the official definition, immune checkpoint inhibitor therapy is considered to be nonresponsive if the TIDE score is higher than 0, while a TIDE score lower than 0 is indicative of a responsive therapy.

Subsequently, we formulated the immunotherapy score (IPS) by detecting the presence of immunosuppressive transmembrane proteins, PD-1 and CTLA4, on the surface of T cells in GC patients. The TCGA-STAD immunotherapy scoring file can be accessed from the TCIA website (https://tcia.at/). By dividing the expression of PD-1 and CTLA4 into four groups (PD-1 positive/CTLA4 negative, PD-1 negative/CTLA4 positive, PD-1 positive/CTLA4 positive, and PD-1 negative/CTLA4 negative), we aimed to investigate the immune prognostic signatures (IPS) in gastric adenocarcinoma between high- and low-risk groups. We further investigated the differences in PD-1, PD-L1, and CTLA4 levels between gastric adenocarcinoma and normal patients using the “ggpubr” and “ggplot2” R packages.

### 2.4. Gene Enrichment and Function Analysis Gene Set Enrichment Analysis (GSEA)

An analysis of Gene Ontology (GO) and Kyoto Encyclopedia of Genes and Genomes (KEGG) pathways was conducted on mRNAs related to ferroptosis, which exhibited varying expression levels, in order to gain insight into the mechanism that distinguishes between high- and low-risk groups of gastric adenocarcinomas. A potential gene set pathway was identified, with an FDR of less than 0.05. GSEA (version 4.0.3) was employed to assess the enrichment degree and statistical significance of a ferroptosis-related gene set between two groups [32]. This algorithm was used to explore the potential functions and pathways of the signature genes. It was determined that a 25% FDR and a nominal *p* value of less than 0.05 would be the threshold for significance.

### 2.5. Consensus Clustering Analysis and GSVA (Gene Set Variation Analysis) Analysis

Utilizing the “ConsensusClusterPlus” R package, we clustered the TCGA-STAD cohort into four groups based on the consensus expression of the lncRNAs associated with ferroptosis [33]. To achieve consensus clustering, 1000 k-means iterations were conducted, and 80% of the genes or samples were bootstrapped. Subsequently, the Kaplan–Meier statistics were applied to analyze the differences in OS across clusters. GSVA analysis revealed the activation status of genes related to ferroptosis and the associated biological pathways [34]. We graphically represented these biological processes using heatmaps, with red representing activation and blue representing inhibition.

### 2.6. The Ferroptosis Potential Index (FPI) Model

The functional profile index (FPI) is a metric for assessing the level of ferroptosis and its significance. ssGSEA was employed to calculate the gene set enrichment scores (ES) which either stimulate or inhibit ferroptosis. Analyses of gastric adenocarcinoma samples showed that a higher FPI score is usually associated with metastasis, medical characteristics, and drug responsiveness [35]. This model enabled us to evaluate the FPI value in each gastric adenocarcinoma sample, thereby uncovering the ferroptosis level of each patient.

### 2.7. Drug IC_50_ Prediction and Secondary Structure Prediction

We established the score of related immune cells by comparing the immune cells of high- and low-risk groups. The Genomics of Drug Sensitivity in Cancer (GDSC) database provides an extensive list of drugs (https://www.cancerrxgene.org/) [36]. Utilizing the PRRophetic algorithm, we established a ridge regression model. The “PRRophetic” R package was utilized to forecast the IC_50_ values of high- and low-risk groups in TCGA-STAD [37]. Twelve antitumor drugs were evaluated, and the IC50 values between the two groups varied significantly. A drug with a lower IC50 was found to be more effective in inhibiting cancer cells. Additionally, we investigated the steadiness of lncRNA structure on the lnCAR database (https://lncar.renlab.org/website), acquiring the secondary structure of LINC00460 and miR205HG [38]. The stability of the lncRNA secondary structure is indicative of the structure and purpose of RNA. LINC00460 and miR205HG were acquired showing a comparatively consistent secondary structure.

### 2.8. Gene Mutation and m6A RNA Methylation Regulator

A comprehensive analysis of gene mutation was conducted using the maftools R package, and gastric adenocarcinomas were divided into high- and low-risk groups. Additionally, mutations of ferroptosis-related mRNAs were also evaluated. We conducted a Pearson analysis to investigate the correlation between CDKN2A and the five related lncRNAs, given that the mutation pathway of cyclin-dependent kinase inhibitor 2A (CDKN2A) in gastric adenocarcinoma was the most significant. We obtained the mutation maf file of each gastric adenocarcinoma patient from the TCGA database, which enabled us to calculate the TMB score of each patient. By analyzing the mutations of gastric adenocarcinoma and based on the median score, we divided the samples into high- and low-mutation groups. We employed the “reshape2” and “limma” R packages to analyze the expression of m6A RNA methylation regulators between the high- and low-risk groups.

### 2.9. Statistical Analysis

The data processing for this study was done using R software (version 4.0.3; https://www.R-project.org). The decision curve analysis (DCA) and operating characteristic curve (ROC) were employed to investigate the sensitivity, specificity, and accuracy of the prognostic features of STAD by leveraging “timeROC” and “ggDCA” packages, respectively. This was done in comparison to other clinicopathological characteristics. Utilizing the Kaplan–Meier survival analysis, the overall survival of STAD patients was evaluated in terms of the ferroptosis-related lncRNA signatures. All analyses yielded a *p* value of less than 0.05, indicating a statistically significant difference.

## 3. Results

### 3.1. Identification of Ferroptosis-Related Differentially Expressed mRNAs and lncRNAs and Construction of a PPI Network

**Figure 1:**
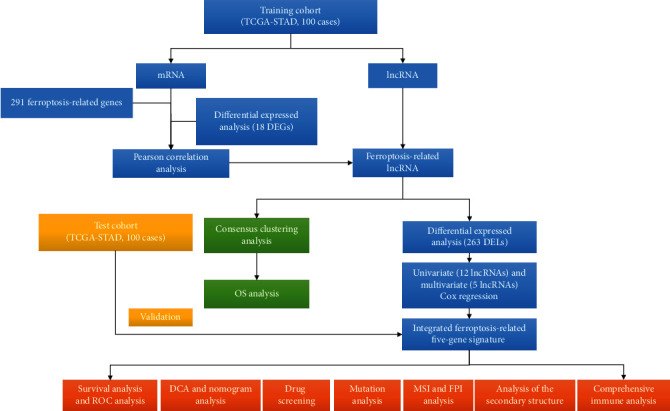
A flow chart of overall study design.

**Figure 2:**
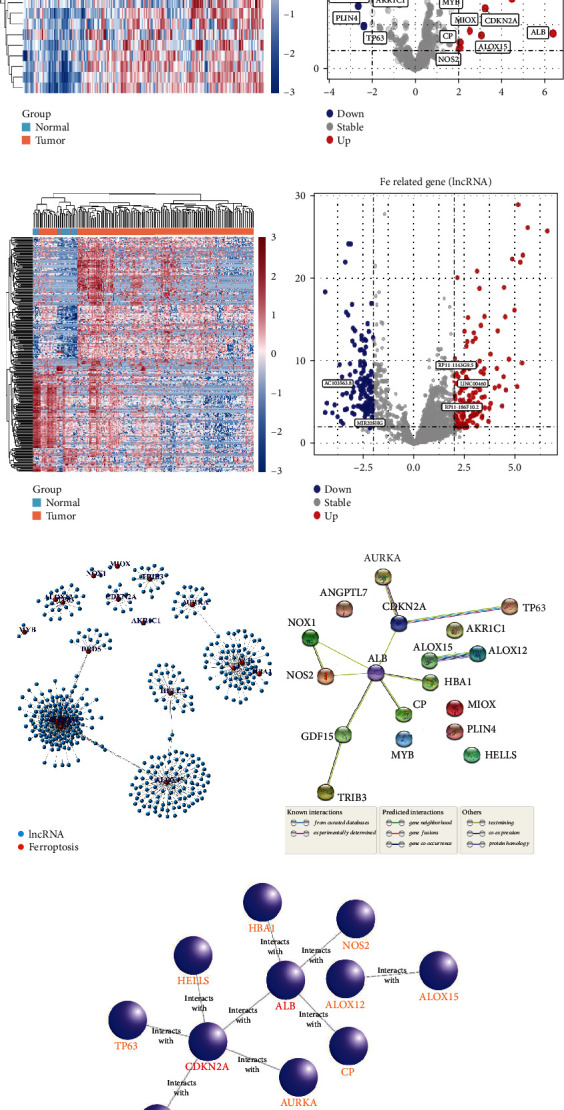
Screening of differentially expressed ferroptosis-related lncRNAs in gastric adenocarcinoma. (a) Heatmap of ferroptosis-related mRNA expression in gastric adenocarcinoma and normal tissues. (b) Volcano plot manifesting differentially expressed genes (DEGs) in ferroptosis-related mRNAs. (c) Heatmap of ferroptosis-related lncRNA expression in gastric adenocarcinoma and normal tissues. (d) Volcano plot manifesting DEGs in ferroptosis-related lncRNA. (e) The correlated network between 263 ferroptosis-related lncRNAs and 18 mRNAs. (f) Differentially expressed ferroptosis-related genes in PPI. (g) The selected 12 hub genes of ferroptosis-related genes via Cytoscape.

As depicted in Figure 1, we combined data from TCGA, FerrDb, and Gene Card databases to construct gene matrices associated with ferroptosis. We utilized the “edgR” package to conduct a differential analysis of 18 ferroptosis-related genes with significant differential expression in gastric adenocarcinoma, resulting in 12 upregulated genes (ALB, ALOX15, GDF15, CDKN2A, HELLS, MIOX, TRIB3, AURKA, NOX1, CP, NOS2, and MYB) and 6 downregulated genes (ANGPTL7, PLIN4, ALOX12, TP63, HBA1, and AKR1C1) (Figures 2(a) and 2(b)). To identify lncRNAs associated with ferroptosis, a Pearson correlation analysis (with Pearson *R* > 0.5 and *p* < 0.001) was performed, resulting in 142 upregulated and 121 downregulated ferroptosis-related lncRNAs (Figures 2(c) and 2(d)). The correlation between ferroptosis genes and lncRNAs was depicted in Figure 2(e), and a PPI network in the STRING database showed the relationship between 18 nodes and 11 edges (Figure 2(f)). In addition, 12 hub genes were further pinpointed by the CytoHubba application (Figure 2(g)).

### 3.2. Establishment of Ferroptosis-Related lncRNA Prognostic Signature

**Figure 3:**
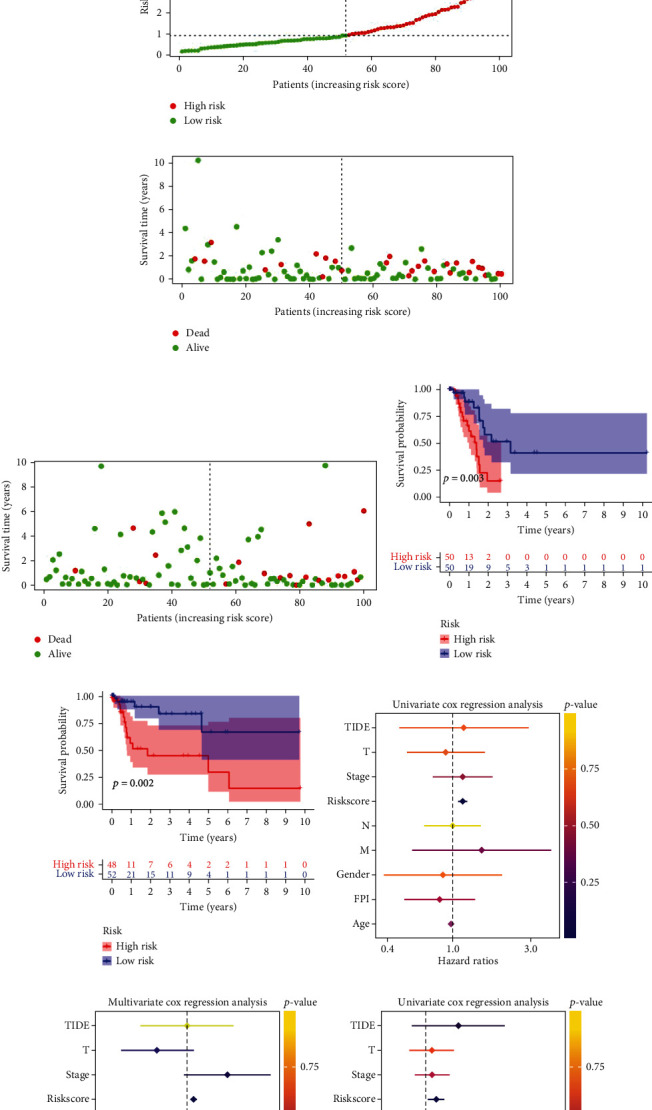
Construction and evaluation of a ferroptosis-related lncRNA prognostic signature in training and test sets. (a, b) Clinical characteristics of gastric adenocarcinoma patients in TCGA training and test sets. (c–f) Distribution of risk score, survival status, and (g–h) OS Kaplan–Meier curves (training cohort: *p* = 0.003, test cohort: *p* = 0.002) of STAD patients in TCGA training and test cohorts. (i–l) The independence of the ferroptosis-related lncRNA signature in OS was verified by univariate and multivariate Cox regression analysis in TCGA training cohort.

**Figure 4:**
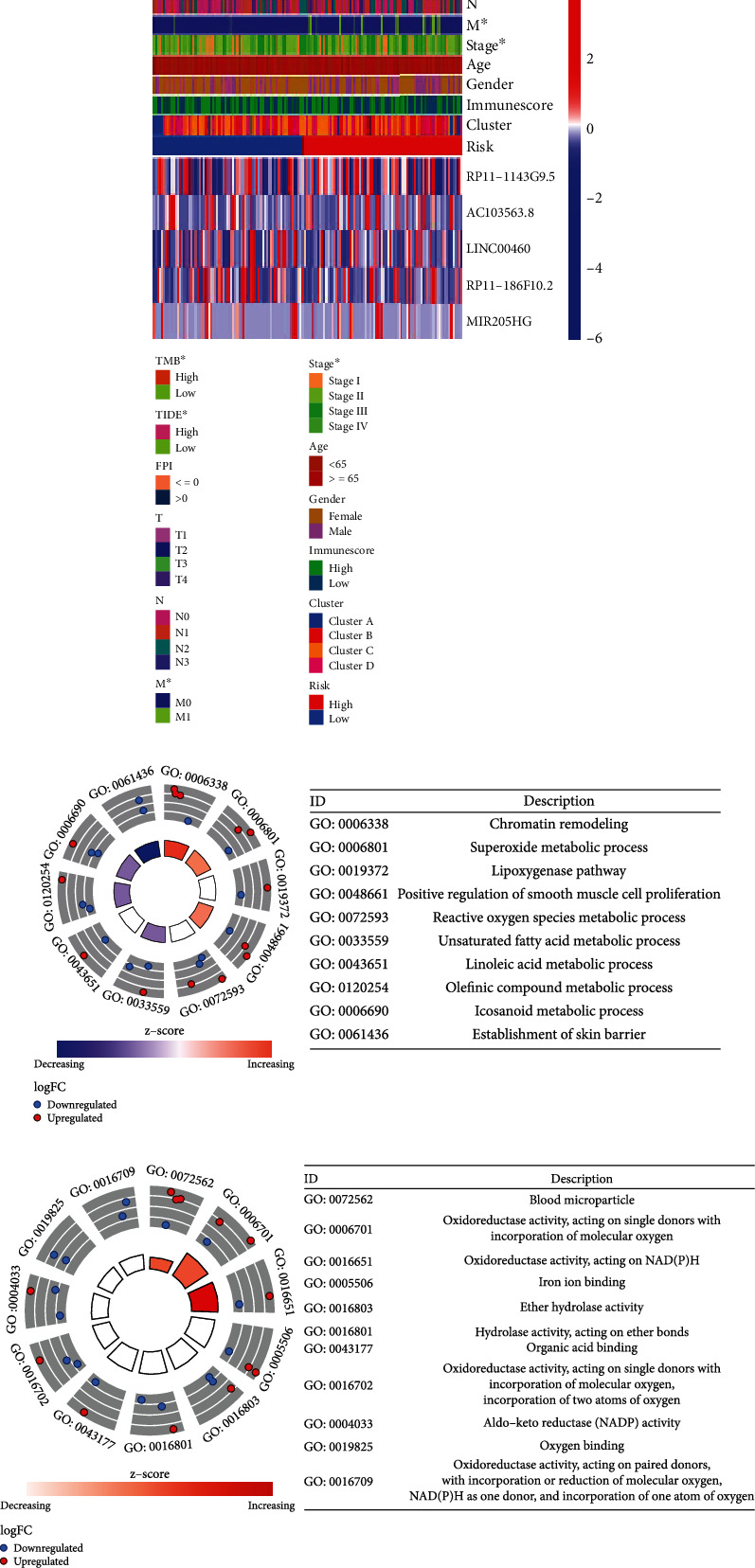
Screening of candidate genes and functional enrichment analysis. (a) Selection of ferroptosis-related lncRNAs related to prognosis by univariate Cox regression analysis. (b) Five ferroptosis-related lncRNAs correlated to the prognosis of gastric adenocarcinoma were obtained by multivariate Cox regression analysis. (c) Expression in different clinical characteristics (including TMB, TIDE, FPI, TNM stage, stage, age, gender, immune score, cluster, and risk) of five ferroptosis-related lncRNAs in gastric adenocarcinoma. (d, e) Gene Ontology (GO) analysis. (f) Kyoto Encyclopedia of Genes and Genomes (KEGG) pathway analysis.

We conducted a screening of 200 TCGA-STAD samples with complete clinical information and randomly allocated them into two groups, with 100 samples in each group for training and testing purposes (as depicted in Figures 3(a) and 3(b)). The samples were divided into two groups, a high-risk group and a low-risk group, based on the median value of the risk score, in order to assess the prognosis of gastric adenocarcinoma patients. The risk score and survival status distribution are illustrated in Figures 3(c)–3(f). Survival analysis revealed that the high-risk group had a worse survival rate in the training set (*p* = 0.003) and test set (*p* = 0.002) (Figures 3(g) and 3(h)). To gauge whether the ferroptosis-related lncRNA signature acted as an independent prognostic factor in STAD patients, univariate and multivariate Cox regression analyses were employed, taking into account TIDE, TNM, stage, risk score, gender, age, and FPI (Figures 3(i)–3(l)). The results of the study confirmed that the risk score was a significant prognostic factor (*p* < 0.001). Univariate Cox regression analysis identified 12 ferroptosis-related lncRNAs (RP11-186F10.2, RP4-781 K5.5, LINC01537, LINC00601, AC103563.8, AC103563.9, RP11-1143G9.5, LINC00460, RP11-64B16.4, LINC00454, KB-68A7.1, and miR205HG) as having a strong association with the prognosis of gastric adenocarcinoma patients, as determined by the “survival” R package. Multivariate Cox regression analysis was used to screen for biomarkers in relation to the prognosis of patients, and 5 ferroptosis-related lncRNAs (RP11-1143G9.5, AC103563.8, LINC00460, RP11-186F10.2, and miR205HG) were selected (Figures 4(a) and 4(b), Supplementary Table 1 and Supplementary Table 2). Additionally, a ferroptosis-related lncRNA signature and the clinical features associated with this signature were evaluated and constructed, including TMB, TIDE, FPI, TNM stage, stage, age, gender, immune score, cluster, and risk (Figure 4(c)). A prognostic model of gastric adenocarcinoma was established with these five ferroptosis-related lncRNAs. We then established a prognostic risk score for the 5 ferroptosis-related lncRNAs, and the risk score was equal to the following: (expression value of RP11 − 1143G9.5 × (−0.423917081201679)) + (expression value of AC103563.8 × 1.2071928010986) + (expression value of LINC00460 × 0.40956647259787) + (expression value of RP11 − 186F10.2 × 1.07891999972853) + (expression value of miR205HG × (−0.366418490206812)).

### 3.3. Validation of the Ferroptosis-Related lncRNA Signature

A nomogram was created to assess the precision and dependability of the prognostic model, incorporating clinical characteristics such as age, tumor (T) status, metastasis (M) status, risk score, stage, and risk, as well as 1-, 3-, and 5-year calibration curves (see Figures S1A and S1B). Results from the examination of the relationship between microsatellite instability and risk score demonstrated that microsatellite stability (MSS) had a more significant influence on the high-score group (70%) than on the low-score group (62%), as shown in Figure S1C and S1D. Figure S1E–G displays the DCA curves of 3, 5, and 7 years. The DCA curves demonstrated that the features associated with the ferroptosis-related lncRNA signature had a superior predictive value. The Kaplan–Meier curves of the five ferroptosis-related lncRNAs (AC103563.8, LINC00460, miR205HG, RP11-186F10.2, and RP11-1143G9.5) between the high- and low-risk groups are shown in Figure S2A–E. Except for RP11-1143G9.5, the OS of the other four genes (AC103563.8, LINC00460, miR205HG, and RP11-186F10.2) in the high-risk group was significantly lower than that in the low-risk group. Additionally, we explored the expression of these five ferroptosis-related lncRNAs in various tissues and organs (Figure S3A–E). Among them, RP11-1143G9.5, RP11-186F10.2, and AC103563.8 were expressed in gastric tissues. It is worth mentioning that AC103563.8 and RP11-1143G9.5 were highly expressed in gastric tissues compared with other tissues and organs. The ROC curves of 3, 5, and 7 years reflected the advantage of the model, which included all sets (3 years, AUC = 0.754; 5 years, AUC = 0.707; 7 years, AUC = 0.797), training set (3 years, AUC = 0.874; 5 years, AUC = 0.786; 7 years, AUC = 0.786) and test set (3 years, AUC = 0.753; 5 years, *AUC* = 0.682; 7 years, AUC = 0.737) (Figure S4A–I).

### 3.4. Gene Enrichment and Function Analysis

We further investigated the biological functions of DEGs by utilizing the “clusterProfiler,” “org.Hs.eg.db,” and “enrichplot” R packages for GO annotation and KEGG pathway analysis. The *p* filter and *p* adjust filter had a value of less than 0.05, respectively. This study conducted GO pathway and process enrichment analysis, which included molecular function (functional set), biological process (pathway), and cellular component (structural complex). The top 21 clusters and their representative enrichment terms are shown in Figures 4(d) and 4(e). The consequence of GO functional annotation demonstrated that the biological processes related to oxygen metabolism were significantly correlated with the differential expression of ferroptosis-related genes, including GO:0006801 (superoxide metabolic process), GO:0019372 (lipoxygenase pathway), GO:0072593 (reactive oxygen species metabolic process), GO:0016701 (oxidoreductase activity, acting on single donors with incorporation of molecular oxygen), GO:0016651 (oxidoreductase activity, acting on NAD(P)H), GO:0016702 (oxidoreductase activity, acting on single donors with incorporation of molecular oxygen, incorporation of two atoms of oxygen), GO:0019825 (oxygen-binding activity), and GO:0016709 (oxidoreductase activity, acting on paired donors, with incorporation or reduction of molecular oxygen, NAD(P)H as one donor, and incorporation of one atom of oxygen). In addition, the ferroptosis-related genes were also related to cell proliferation, such as GO:0048661 (positive regulation of smooth muscle cell proliferation). KEGG pathway enrichment analysis showed that the ferroptosis pathway was significantly enriched (Figure 4(f)), which mechanism and regulation of intracellular Fe^2+^ as shown in Figure S5A and S5B. GSEA for the ferroptosis-associated lncRNA signature demonstrated that gene silencing, negative regulation of gene expression, and posttranscriptional regulation of gene expression were significantly enriched in high-risk groups of gastric adenocarcinoma samples (Figure S5C–G).

### 3.5. Consensus Clustering Analysis of Ferroptosis-Related lncRNAs and GSVA Analysis

**Figure 5:**
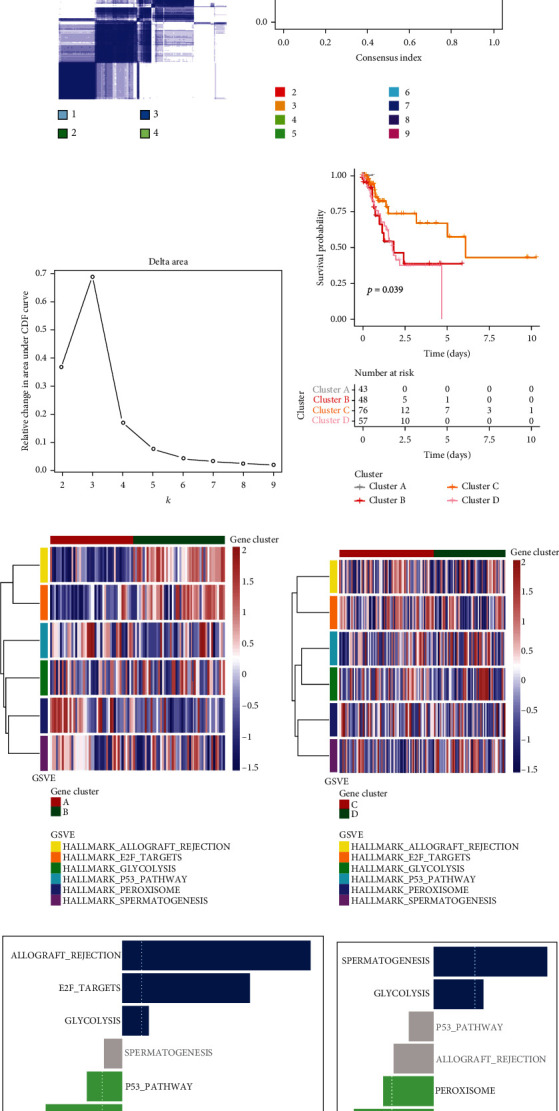
The OS of STAD in the cluster A/B/C/D subgroups and GSVA enrichment analysis. (a) Consensus clustering matrix for *k* = 4. (b, c) When *k* = 2–9, the consensus clustering cumulative distribution function (CDF) and relative change of the area under the CDF curve. (d) Kaplan–Meier curves of the overall survival for patients with STAD in four clusters (cluster A/B/C/D). (e–h) GSVA enrichment analysis showed the activation status of ferroptosis-related genes and related biological pathways. Heatmaps are used to visualize these biological processes, with red indicating activation pathways and blue indicating inhibition pathways. The GC cohort was annotated as a sample. (e, g) Cluster A vs. cluster B. (f, h) Cluster C vs. cluster D.

The “ConsensusClusterPlus” R package was used to cluster the ferroptosis-related lncRNAs into four clusters, and the crossover between STAD samples was found to be the lowest in this case (Figures 5(a)–5(c)). Consequently, we divided the samples into four clusters (A/B/C/D). Compared with other clusters, the Kaplan–Meier algorithm found that the patients in cluster C had a better OS than the patients in cluster A (Figure 5(d)). GSVA enrichment analysis showed the activation status of ferroptosis-related genes and related biological pathways. As shown in Figures 5(e) and 5(f), ferroptosis-related genes were enriched in allograft rejection, E2F targets, glycolysis, p53 pathway, peroxisome, and spermatogenesis. Comparing the enrichment of cluster A and cluster B, the results demonstrated allograft rejection and 8E2F significant enrichment in cluster A, and cluster A was negatively regulated relative to cluster B in the p53 pathway (Figure 5(g)). A comparison between cluster C and cluster D revealed that cluster C had a higher presence of peroxisome and E2F target, as illustrated in Figure 5(h).

### 3.6. Comprehensive Immunoassay of Immune Infiltration, Immune Checkpoints, and Immunotherapy Response

**Figure 6:**
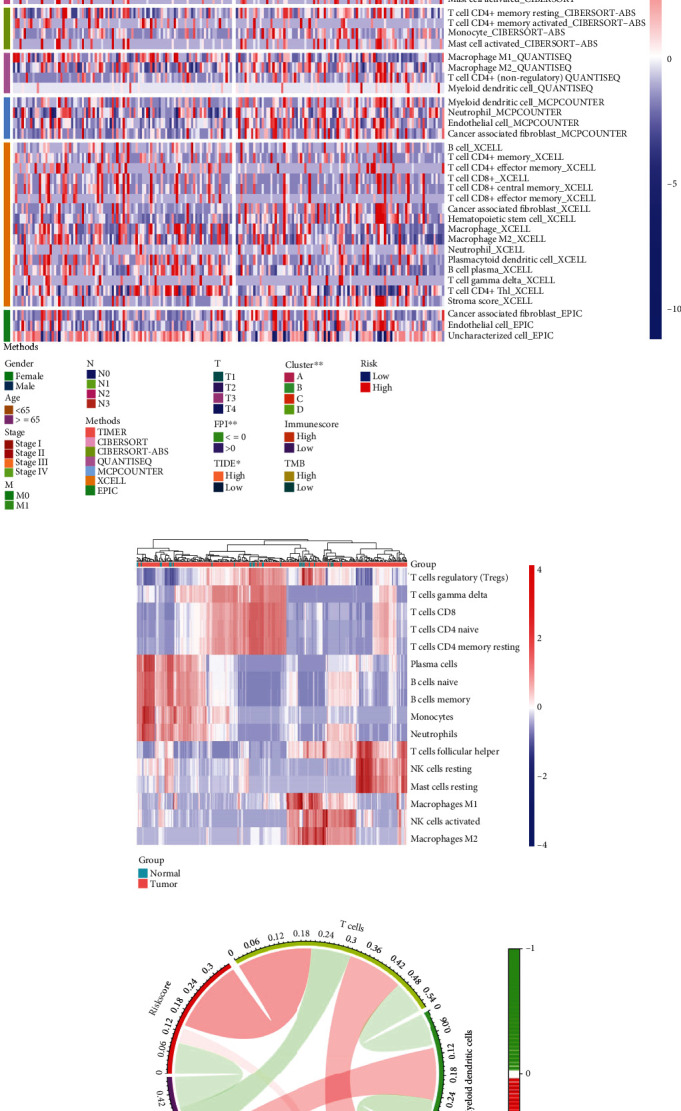
The results of immune cell infiltration were verified based on seven different algorithms. (a) A heatmap based on seven different algorithms (TIMER, CIBERSORT, CIBERSORT-ABS, QUANTISEQ, MCPCOUNTER, XCELL, and EPIC) of the immune infiltration of gastric adenocarcinoma patients with different clinical characteristics in TCGA. (b) The heatmap shows the expression of 16 types of immune cells in tumor and normal tissues. (c) Correlation of T cells, endothelial cells, and myeloid dendritic cells with TMB and risk score. Red represents positive correlation, and green represents negative correlation.

**Figure 7:**
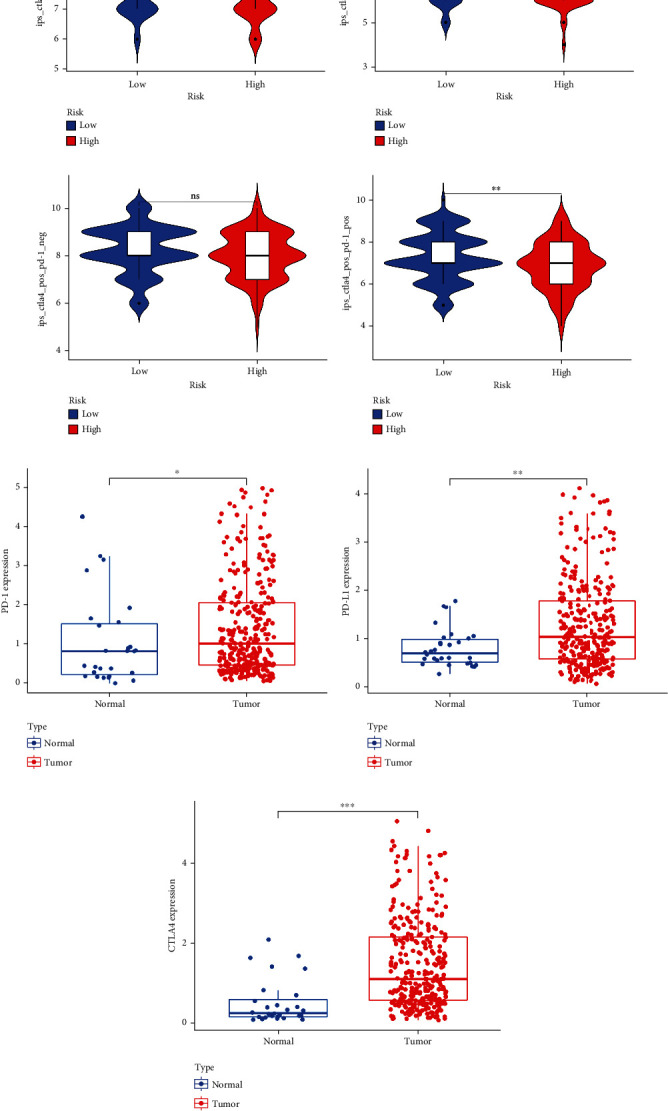
Immunotherapy scores (IPS) and gene expression analysis of immune checkpoints. (a) IPS of CTLA4-negative and PD-1-negative cells in high- and low-risk groups. (b) IPS of CTLA4-negative and PD-1-positive cells in high- and low-risk groups. (c) IPS of CTLA4-positive and PD-1-negative cells in high- and low-risk groups. (d) IPS of CTLA4-positive and PD-1-positive cells in high- and low-risk groups. (e–g) Expression of PD-1, PD-L1, and CTLA4 in the gastric adenocarcinoma and normal groups. The expressions of PD-1, PD-L1, and CTLA4 in tumor tissues were significantly higher than those in normal tissues. ^∗^*p* < 0.05; ^∗∗^*p* < 0.01; ^∗∗∗^*p* < 0.001.

**Figure 8:**
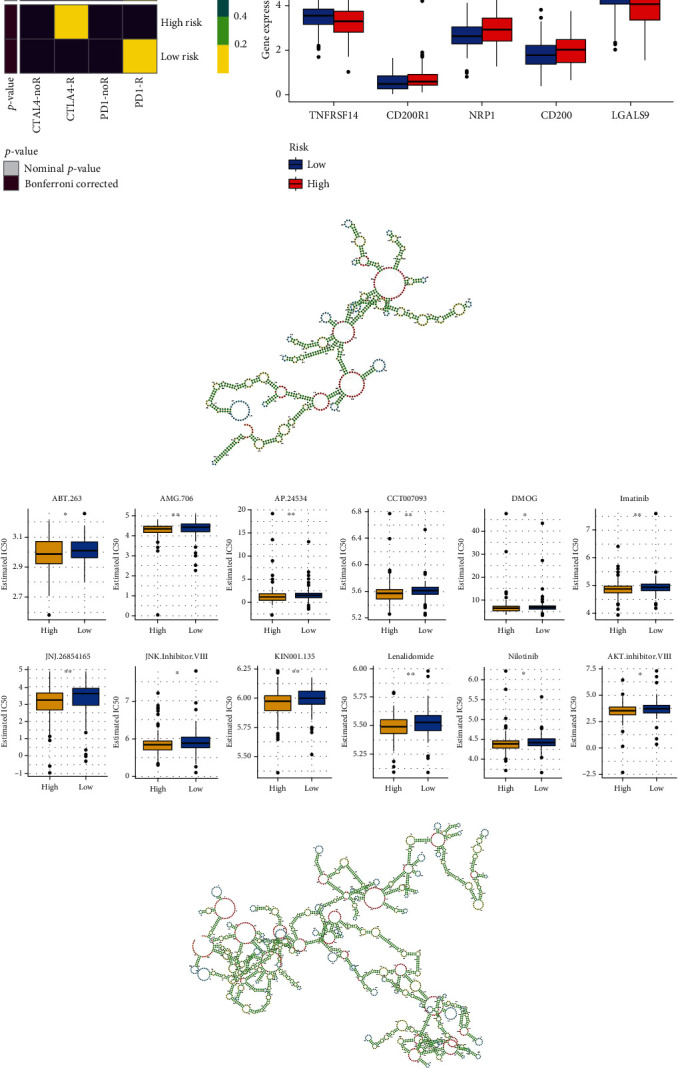
Anti-CTLA4, anti-PD-1 immunotherapy, construction of secondary structure, and drug IC_50_ prediction. (a) The heatmap shows the response of anti-CTLA4 and anti-PD-1 immunotherapy for gastric adenocarcinoma in the high-risk and low-risk groups. In terms of a nominal *p* value, anti-CTLA4 immunotherapy was more likely to lead to a response in the high-risk group (*p* = 0.007), and the Bonferroni corrected *p* value was less than that in other cases. (b) Expression of the five immune checkpoint inhibitors in the high- and low-risk groups. (c) The secondary structure of LINC00460. (d) The IC_50_ values of 12 drugs expressed in the high-risk and low-risk groups were used to screen drugs with substantial differences between the two groups. (e) The secondary structure of miR205HG. IC_50_, half-maximal inhibitory concentration. ^∗^*p* < 0.05; ^∗∗^*p* < 0.01; ^∗∗∗^*p* < 0.001.

To gain a better comprehension of the relationship between risk scores and immune cells, we conducted an analysis of immune cells between the high- and low-risk groups of TCGA-STAD, utilizing seven algorithms. The heatmap plot presented the expression of immune cells in both the high- and low-risk groups, as well as various clinical features (Figure 6(a)). Cancer-associated fibroblasts, hematopoietic stem cells, stroma score, B cells, T cell CD4+ memory, T cell CD4+ effector memory, CD8+ T cells, and T cell CD8+ central memory were all significantly expressed in the high-risk group, with *p* values of less than 0.01, 0.001, 0.05, 0.05, 0.05, 0.05, 0.05, and 0.01, respectively. Tumor tissues exhibited a notable rise in the expression of T follicular helper cells, resting NK cells, and resting mast cells (Figure 6(b)). Correlation analysis was used to show the interaction among immune cells, risk score, and TMB. The results demonstrated that T cells, endothelial cells, and myeloid dendritic cells had positive and negative regulatory relationships with a risk score and TMB. As depicted in Figure 6(c), red was indicative of a positive correlation, whereas green was indicative of a negative correlation. The ESTIMATE score displayed an inverse correlation with tumor purity, as demonstrated in Figure S6A. In addition, we analyzed the score of connected immune cells and immune-related pathways in the high- and low-risk groups (Figure S6B). The proportion of 22 immune cells in gastric adenocarcinoma samples was manifested by heatmap and box plot based on the CIBERSORT algorithm (Figure S6C and 6E). We analyzed the expression levels of 23 regulatory factors linked to m6A between high- and low-risk groups. It was noteworthy that the expression of FTO (alpha-ketoglutarate dependent dioxygenase) (*p* < 0.01), IGFBP3 (insulin-like growth factor-binding protein 3) (*p* < 0.05), and VIRMA (*p* < 0.05) in the high-risk group was higher than that in low-risk group (Figure S6D). Immunotherapy scores were constructed by the expression of PD-1 and CTLA4 in T cells of patients with gastric adenocarcinoma. IPS was evaluated with four groups, including PD-1 negative CTLA4 negative (Figure 7(a)), PD-1 positive CTLA4 negative (Figure 7(b)), PD-1 negative CTLA4 positive (Figure 7(c)), and PD-1 positive CTLA4 positive (Figure 7(d)). The data revealed that the high-risk group had poorer scores than the low-risk group, signifying that their immunotherapy was not as effective. The scoring file of TCGA-STAD immunotherapy was downloaded from the TCIA database. The expression of PD-1, PD-L1, and CTLA4 between tumor tissue and normal tissue was also explored via the “limma” R package. The box plot indicated that expression of PD-1 (Figure 7(e)), PD-L1 (Figure 7(f)), and CTLA4 (Figure 7(g)) in tumor tissues was notably higher than in normal tissues. PD-1 inhibited T cell activation and induced T cell death by binding with PD-L1 (or PD-L2), playing a paramount role in tumor immunotherapy. It demonstrated that the tumor immune escape ability of the high-risk group was stronger than that of the low-risk group. The high-risk group was more likely to respond to anti-CTLA4 immunotherapy, as indicated by a nominal *p* value of 0.007, and its Bonferroni-corrected *p* value was lower than that in other cases (Figure 8(a)). The data from Figure 8(b) indicates that the ICIs connected to the research were expressed in greater amounts in the high-risk group than in the low-risk group.

### 3.7. Prediction of Antitumor Drug and Secondary Structure, Immunotherapy Scores (IPS), Ferroptosis Potential Index (FPI), and Mutation Analysis

**Figure 9:**
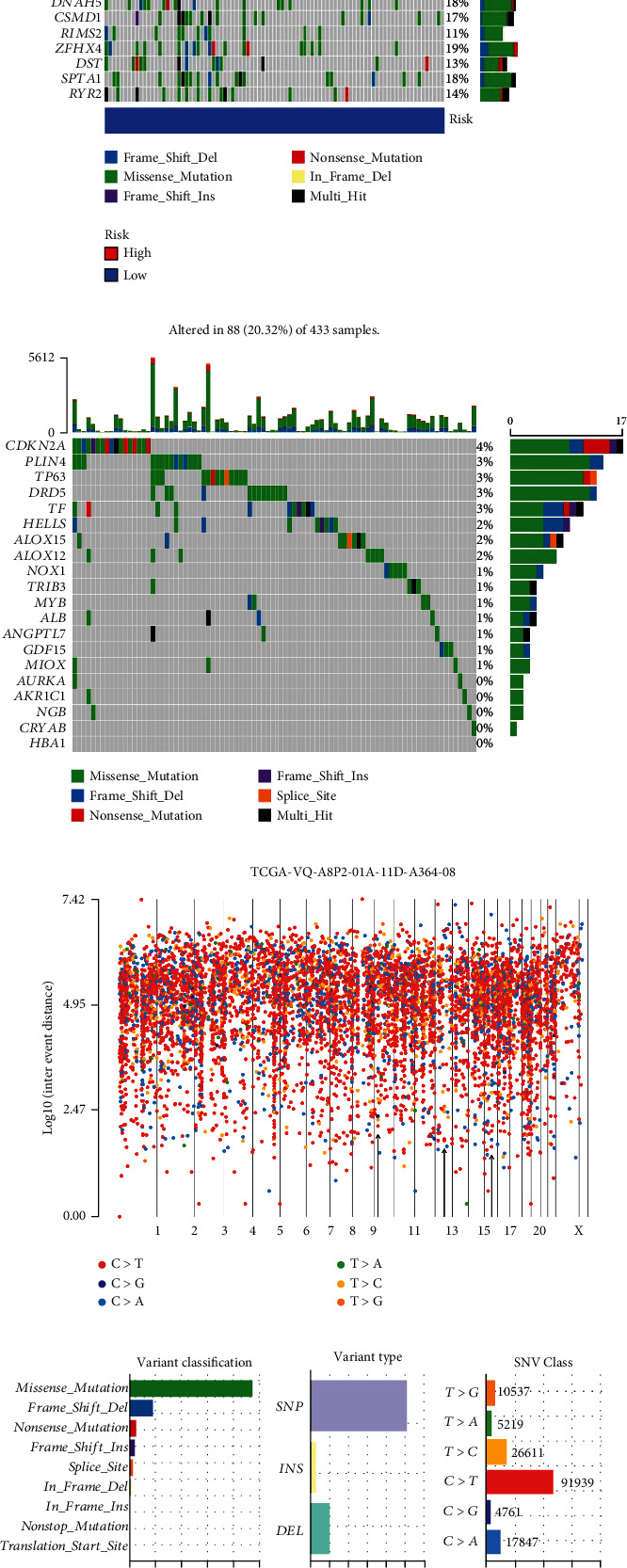
Landscape of TMB in STAD. (a, b) The top 20 mutant genes with the highest mutation rate in the high- and low-risk groups. (c) Mutations of ferroptosis-related genes in STAD patients. (d) Chromosome mutation in gastric adenocarcinoma samples. (e) A comprehensive plot of genetic alterations in STAD. *C* > *T* occurred frequently in single nucleotide variation (SNV). (f) The relevance between highly mutated gene CDKN2A and a five ferroptosis-related lncRNA signatures (*R* > 0.1, *p* < 0.05). ^∗^*p* < 0.05; ^∗∗^*p* < 0.01; ^∗∗∗^*p* < 0.001.

As shown in Figures 8(c) and 8(e), LINC00460 (MFE = −273.3 kcal/mol) and miR205HG (MFE = −1109.3 kcal/mol) both showed stable secondary structure, reflecting the function of RNA transcription. We established a Ridge regression model for forecasting the IC_50_ of drugs. The *p* value of all 12 drugs (ABT.263, AMG.706 (motesanib), AP.24534, CCT007093, DMOG, imatinib, JNJ.26854165, JNK inhibitor VIII, KIN001.135 (benzimidazole-thiophene carbonitrile), lenalidomide, and nilotinib, AKT inhibitor VIII.) were less than 0.05 (Supplementary Table 3).  Figure 8(d) demonstrated that the IC_50_ value for the high-risk group was greater than that for the low-risk group, demonstrating enhanced antitumor effectiveness. To gain a better understanding of the role of ferroptosis-related lncRNAs in gastric adenocarcinoma, the secondary structure of the five lncRNA biomarkers was determined using the lnCAR database. We defined an FPI less than or equal to 0 as the low-score group and an FPI greater than 0 as the high-score group. After removing meaningless samples, we included 198 gastric adenocarcinoma samples and divided them into four groups, including (A) “FPI < 0 high-risk” group, (B) “FPI < 0 low-risk” group, (C) “FPI > 0 high-risk” group, and (D) “FPI > 0 low-risk” group (Figure S7A). The Kaplan–Meier analysis concluded that the (D) “FPI > 0 low-risk” group had the best OS, and “FPI < 0 low-risk” (B) had the worst OS. The findings of the study suggested that a high FPI score can reduce the risk of gastric cancer. A total of 198 cases of gastric adenocarcinoma were studied, out of which 122 had an FPI score greater than 0 (Figure S7B), and 76 had an FPI score less than 0 (Figure S7C). The Kaplan–Meier analysis showed that the patients with an FPI score greater than 0 had a worse survival rate in the high-risk group (*p* = 0.0328). Moreover, the high and low FPI scores were evaluated in the context of MSI (microsatellite instability) analysis. Figures S7D and S7E illustrate that the high-risk group, as determined by their FPI score, had a greater prevalence of MSI-H (17%) than the low-risk group (15%). Tumor tissues had a considerably greater FPI than normal tissues (*p* = 8.3*e* − 17), as illustrated in Figure S7F. In order to explore the mutations of ferroptosis-related genes in STAD, a mutation panorama of STAD genes was analyzed between the high-risk group and low-risk group (Figures 9(a) and 9(b)). TTN, TP53, MUC16, SYNE1, and LRP1B were found to be highly mutated in both the high-risk and low-risk groups. The highest mutation rate of ferroptosis-related genes in gastric adenocarcinoma samples was CDKN2A (Figure 9(c)). The results demonstrated that there was a significant correlation between CDKN2A and LINC0046 (*R* > 0.1, *p* < 0.05). The *C* > *T* occurred frequently in single nucleotide variation (SNV) (Figures 9(d) and 9(e)). The Pearson analysis was performed between CDKN2A and five ferroptosis-related lncRNA biomarkers (Figure 9(f)). RTK-RAS, Hippo, and TP53 pathways were vulnerable to STAD gene mutations (Figure S8A). The erb-b2 receptor tyrosine kinase 4 (ERBB4) showed a high mutation state in the RTK-RAS pathway (Figure S8B). Notably, CUL3 was the most highly mutated gene in the NRF2 pathway (Figure S8C). The mutation rate of serine/threonine kinase (ATM) was higher in the TP53 pathway (Figure S8D). In the NOTCH pathway, the mutation rate of contactin 6 (CNTN6) was the highest (Figure S8E), and in the PI13K pathway, PIK3CA was the most highly mutated gene. (Figure S8F). In the Hippo pathway, both FAT atypical cadherin 3 (FAT3) and FAT atypical cadherin 4 (FAT4) showed high mutation rates (Figure S8G). Interestingly, the highest mutated gene in the cell cycle pathway was CDKN2A, which was consistent with our previous research conclusions (Figure S8H). ACVR2A and APC were observed to have high mutation rates in the TGF-beta and WNT pathways, respectively, as depicted in Figure S8I–J.

## 4. Discussion

As a novel modality of iron-dependent cell death characterized by the accumulation of lipid peroxides and reactive oxygen species, ferroptosis presents an innovative viewpoint on the treatment of cancer and provides the possibility of developing new strategies for the treatment of gastric adenocarcinoma [8, 9]. Increased ferroptosis has been shown to aid in the anticancer effectiveness of immunotherapy, according to a recent research [39], which suggests a strong association between ferroptosis and immunotherapy. Despite the lack of research on the role of ferroptosis-related lncRNAs in gastric adenocarcinoma, particularly the mechanism connecting it to the immune microenvironment, our study seeks to address this issue. We conducted a thorough investigation of ferroptosis-related lncRNAs in gastric adenocarcinoma, which included evaluating immunotherapy response, immune infiltration, IPS scores, predicting somatic mutations, analyzing tumor immune microenvironment, and assessing tumor drug sensitivity, as well as analyzing the stability of the secondary structure of lncRNAs in RNA transcription.

In this study, we conducted a comprehensive analysis of the TCGA database to identify ferroptosis-related lncRNAs and assess their potential predictive value. After differential analysis, 18 mRNAs and 263 lncRNAs were found to be differentially expressed between gastric adenocarcinoma and normal tissues. Subsequently, a univariate Cox regression analysis was employed to identify lncRNA signatures associated with prognosis. Through multivariate Cox regression analysis, we identified five lncRNAs (LINC00460, miR205HG, AC103563.8, RP11-186F10.2, and RP11-1143G9.5) that were associated with overall survival and used them to construct a risk model. In the following analysis, the outcome of the Kaplan–Meier analysis suggested that the high expression of LINC00460, miR205HG, AC103563, and RP11-186F10.2 was strongly associated with poor prognosis in gastric adenocarcinoma tissues, and in turn, RP11-1143G9.5 high expression was closely related to a good prognosis. In addition, we found that the survival rate of patients in the low-risk group was significantly improved, indicating that the low-risk score was closely related to longer survival. In addition, the ROC graph showed that high AUC values amongst the TCGA training set, TCGA training test set, and TCGA entire set for 3, 5, and 7 years were all greater than 6.5, indicating good prediction performance of our model. We also evaluated the clinical characteristics of ferroptosis-related lncRNAs in gastric adenocarcinoma patients in TCGA training and test sets.

The findings of the GO analysis indicated a tight relationship with the principal molecular function of ferroptosis regulation, such as the superoxide metabolic process and iron ion binding. KEGG pathway analysis showed the four important pathways—ferroptosis, arachidonic acid metabolism, serotonergic synapse, and arginine biosynthesis. To assess the correlation of genes related to ferroptosis, we amalgamated the STRING online database and Cytoscape software to create a PPI network comprising 18 nodes and 11 edges. The 10 most important hub genes were identified by the Cytoscape plugin CytoHubba, including ALB, CDKN2A, NOS2, GDF15, NOX1, HBA1, ALOX12, ALOX15, AURKA, and CP. The CDKN2A gene showed an important position in the network. We also summarized genetic aberrations, including the incidence of somatic mutations and copy number variations (CNVs) of 18 ferroptosis regulators in gastric adenocarcinoma. We discover that CDKN2A exhibited the highest mutation frequency, which may improve our understanding of the genetic heterogeneity in GC. Among single nucleotide variations, *C* > *T* was the most commonly encountered. The cell cycle was affected by gene mutations in CDKN2A. CDKN2A was shown previously to be a novel ferroptosis driver [40] and encodes the ARF (alternative reading frame) protein [41]. It has been previously demonstrated that cyclin-dependent kinase inhibitor 2A (CDKN2A/ARF) causes cancer cells to be sensitive to ferroptosis by inhibiting the ability of NRF2 and its transcriptional target SLC7A11 [42] through pathways dependent or independent of p53 tumor suppression gene in the cancerous cells [43, 44]. Therefore, we examined the correlation between CDKN2A, the five lncRNAs, and overall survival (LINC00460, miR205HG, AC103563.8, RP11-186F10.2, and RP11-1143G9.5). Data analysis revealed a substantial correlation between LINC00460 and CDKN2A, implying that these two elements may be related to the survival and prognosis of patients with gastric adenocarcinoma.

LINC00460 is a novel lncRNA with 935 nucleotides located on chromosome 13q33.2. Growing research indicates that the lncRNA LINC00460 has an oncogenic function in the advancement of many malignancies. It has been confirmed that LINC00460 functioned as an oncogene regulating prostate cancer progression through the promotion of cell proliferation and a reduction in apoptosis [45]. Wang et al. demonstrated that LINC00460 can promote the proliferation and repress the apoptosis of non-small cell lung cancer cells by targeting miR-539 [46]. By sponging miR-4443, LINC00460 promotes cell progression in squamous cell carcinoma of the head and neck [47]. By competitively binding miR-489-5p to elevate FGF7 expression and enhancing downstream AKT signaling, LINC00460 promotes breast cancer progression [48]. LINC00460 can also function as a molecular sponge to adsorb miR-1224-5p, thereby promoting esophageal cancer (ESCA) metastasis and progression [49]. Yuan et al. reported that the downregulation of LINC00460 inhibits colorectal cancer metastasis via WWC2 [50]. miR205HG, also known as LINC00510, is a novel lncRNA with 4173 bp located at chromosome location 1q32.2. According to many studies, miR205HG was affirmed as important in its oncogenic role in cancer progression. It has been shown that lncRNA miR205HG drives the advancement of esophageal squamous cell carcinoma through the miR-214/SOX4 axis. [51]. Liu et al. concluded that miR205HG expedited the cell proliferation and progression of lung squamous cell carcinoma via targeting miR-299-3p [52]. miR205HG also functions as a competing endogenous RNA (ceRNA) to accelerate tumor growth and progression via sponging miR-122e5p in cervical cancer [53]. The results demonstrate that expression of lncRNA LINC00460 is higher in tumors, and those with a decrease in LINC00460 expression had a prolonged survival time. In comparison to lncRNA LINC00460, the expression of lncRNA miR205HG was lower in tumor samples than in normal stomach tissue, and those with a decrease in miR205HG had a poorer prognosis. Additionally, the strong association between lncRNA LINC00460 with CDKN2A ferroptosis drivers suggested the potential that LINC00460 affected patient survival via CDKN2A.

It is widely accepted that epithelial–mesenchymal transition is a fundamental mechanism in the progression of cancer cells to invasion and metastasis [54]. lncRNAs can be employed to forecast the clinical course, aggressiveness, invasion, and metastasis potential of GC, in addition to providing a model for investigating ways to inhibit or reverse metastatic potential. lncRNAs can be considered crucial elements of epigenetic regulation of gene expression in tumorigenesis and carcinogenesis, and their impact on the EMT can be achieved by either directly regulating the vimentin and E-cadherin function or by controlling the transcription of these genes through various factors [55]. To further explore the biological nature of the different ferroptosis subtypes, we conducted a gene set variation analysis (GSVA) enrichment. The results showed that four ferroptosis clusters were enriched in the pathway related to immune (allograft rejection), activation of cancer-related pathways (p53 pathway, e2f targets, and peroxisome), cell proliferation-related metabolic (glycolysis), and spermatogenesis, which suggested an important role in tumor progression and a tight association with the tumor microenvironment. An evaluation of immune cell infiltration was conducted using seven distinct algorithms, and it was determined that T cells and macrophages had significantly infiltrated the gastric microenvironment. Previous mouse models of gastritis and GC revealed that a notable quantity of macrophages migrates to the GC microenvironment, which influences angiogenesis or tumor immunity [56–61]. Tumor-infiltrating lymphocytes (TILs) composed of T cells, natural killer (NK) cells, B cells, and T-cell-mediated adaptive immunity are usually considered the manifestation of the host antitumor immune response. The upregulation of PD-L1 or CTLA-4 expression can mediate the escape of tumor cells from the host immune response, lead to an immunosuppressive state, and inhibit the antitumor immune response in some tumor microenvironments [62]. Therefore, IPS and gene expression analysis of immune checkpoints were also performed. We examined the expression levels of PD-1 (PDCD1), PD-L1, and CTLA4 (cytotoxic T-lymphocyte associated protein 4) in tumor and normal groups. The expression of PD-1, PD-L1, and CTLA4 in gastric adenocarcinoma was all higher than in the normal group, with the expression levels of three common immune checkpoints distinctly upregulated in high-risk cohorts. Additionally, the high-risk group was more likely to respond to anti-CTLA4 immunotherapy (*p* = 0.007). Interestingly, in our immunotherapy analysis, the low-risk group achieved higher CTLA4-negative/PD-1-negative, CTLA4-negative/PD-1-positive, and CTLA4-positive/PD-1-positive scores, indicating that patients with a low-risk score are better candidates for immunotherapy. It may be that high-risk patients had a very serious gastric adenocarcinoma or that lncRNAs were highly expressed, reducing immunotherapy effectiveness. To explore immunotherapy responses, the TIDE algorithm was used to identify significant differences in immunotherapy responses between the high- and low-risk groups (better responses in the low-risk group). The TIDE score will help us better select patients who are more suitable for immune checkpoint suppression therapy, and it will be of interest to test the clinical efficacy of TIDE scores in immune checkpoint suppression treatment decision-making in prospective clinical trials. In addition, 12 antigastric adenocarcinoma drugs were screened according to lncRNA signals associated with iron drop: ABT.263, AMG.706 (Motesanib), AP.24534, CCT007093, DMOG, imatinib, JNJ.26854165, JNK inhibitor VIII, KIN001.135 (benzimidazole-thiophene carbonitrile), lenalidomide, nilotinib, and AKT inhibitor VIII. All of the above drugs have different degrees of antitumor effects and vital clinical significance [63–73].

In conclusion, our study identified five ferroptosis-related lncRNAs (LINC00460, mirR205HG, AC103563.8, RP11-186F10.2, and RP11−1143G9.5) involved in gastric adenocarcinoma that are of great value in predicting OS in gastric adenocarcinoma patients. It was discovered that five ferroptosis-related lncRNAs could be used as significant prognostic biomarkers to forecast long-term survival in gastric adenocarcinoma patients and regulate the proliferation and migration of gastric adenocarcinoma cells and the development of ferroptosis. In addition, we evaluated the clinical signs of FPI, TMB, and tide and completed a thorough examination of immunotherapy and drug prediction. It has been noted that immunotherapy, particularly anti-CTLA4 immunotherapy and 12 antigastric adenocarcinoma drugs, has resulted in a higher survival rate for patients with gastric adenocarcinoma. Therefore, lncRNAs (LINC00460, miR205HG, AC103563.8, RP11-186F10.2, and RP11−1143G9.5) may serve as potential biomarkers of prognostic value in gastric adenocarcinoma. This study had certain limitations, such as the use of public databases for mining and analysis. To improve the dependability of our results and eliminate selection bias, larger population and multicenter clinical trials are required. In order to gain insight into the potential functions of the five ferroptosis-related lncRNAs in gastric cancer, since all the mechanical studies in our work were descriptive, further laboratory experiments were necessary.

## Figures and Tables

**Table 1:** The clinical characteristics of TCGA-STAD.

	Alive (*n* = 173)	Dead with tumor (*n* = 47)	Dead tumor free (*n* = 4)	Total (*n* = 224)	*p* value
Gender^∗^					
Female	72 (41.6%)	9 (19.1%)	1 (25.0%)	82 (36.6%)	
Male	101 (58.4%)	38 (80.9%)	3 (75.0%)	142 (63.4%)	0.016
Age^∗∗∗^					
>65	89 (51.4%)	33 (70.2%)	4 (100.0%)	126 (56.2%)	
≤65	84 (48.6%)	14 (29.8%)		98 (43.8%)	4.9e-20
AJCC stage^∗∗^					
Stage I	33 (19.1%)	8 (17.0%)	1 (25.0%)	42 (18.8%)	
Stage II	86 (49.7%)	7 (14.9%)	1 (25.0%)	94 (42.0%)	
Stage III	39 (22.5%)	19 (40.4%)	2 (50.0%)	60 (26.8%)	
Stage IV	15 (8.7%)	13 (27.7%)		28 (12.5%)	0.002
T (tumor)					
T1	15 (8.7%)	1 (2.1%)		16 (7.1%)	
T2	50 (28.9%)	11 (23.4%)	3 (75.0%)	64 (28.6%)	
T3	94 (54.3%)	29 (61.7%)	1 (25.0%)	124 (55.4%)	
T4	14 (8.1%)	6 (12.8%)		20 (8.9%)	0.151
M (metastasis)^∗∗∗^					
M0	164 (94.8%)	40 (85.1%)	4 (100.0%)	208 (92.9%)	
M1	9 (5.2%)	7 (14.9%)		16 (7.1%)	7.4e-45
N (lymph node)					
N1	74 (42.8%)	12 (25.5%)	1 (25.0%)	87 (38.8%)	
N2	56 (32.4%)	11 (23.4%)	1 (25.0%)	68 (30.4%)	
N3	37 (21.4%)	14 (29.8%)	2 (50.0%)	53 (23.7%)	
N4	6 (3.5%)	10 (21.3%)		16 (7.1%)	0.249

^∗^ *p* < 0.05, ^∗∗^*p* < 0.01, ^∗∗∗^*p* < 0.001.

## Data Availability

The original contributions presented in the study are included in the article/Supplementary Material. Further inquiries can be directed to the corresponding author.

## Supplementary Materials

Supplementary Materials Please see the supplementary material section of the author guidelines for details on the different file types accepted.
