# Supplementary material for: Ferroptosis-Related lncRNAs Act as Novel Prognostic Biomarkers in the Gastric Adenocarcinoma Microenvironment, Immunotherapy, and Chemotherapy
Source: Oxid Med Cell Longev. 2023 May 19;2023:9598783. doi: 10.1155/2023/9598783 (PMC10219779; doi:10.1155/2023/9598783)
Supplement: Supplementary Materials — Please see the supplementary material section of the author guidelines for details on the different file types accepted. [file 9598783.f1.docx]

***Supplementary Materia***

**Supplementary Figures**

**Figure S1. Construction and evaluation of the prognostic nomogram, DCA curves and microsatellite instability.** (A) The prognostic nomogram with clinical characteristics (Age, M, T, risk Score, Stage and risk) for the prediction of 1-, 3- and 5-year overall survival in STAD. (B) The calibration plots in 1-, 3-, and 5-years. (C-D) The distribution of different riskScore in microsatellite instability. The relationship between microsatellite instability and risk Score shows that MSS accounts for more in the high score group (70%) than in the low score group (62%). (E-G) The DCA curves in 1-, 3- and 5-years.

**Figure S2**. Kaplan-Meier survival curves analysis of (A) AC103563.8 (*p =* 0.00262542), (B) LINC00460 (*p =* 5.528e-05), (C) MIR205HG (*p =* 0.04103241), (D) RP11-186F10.2 (*p =* 0.00205138), and (E) RP11-1143G9.5 (*p =* 0.04050869). Except for RP11-1143G9.5, the OS of the other four genes (AC103563.8, LINC00460, MIR205HG, and RP11-186F10.2) in the high-risk group was significantly lower than that in the low-risk group. ^*^*p <* 0.05; ^**^*p <* 0.01; ^***^*p <* 0.001.

**Figure S3. The expression in various tissues and organs of 5 ferroptosis-related lncRNAs.** (A) MIR205HG**.** (B) AC103563.8. (C) RP11-1143G9.5. (D) RP11-186F10.2. (E) LINC00460. The results demonstrated RP11-1143G9.5, RP11-186F10.2, and AC103563.8 were expressed in gastric tissues.

**Figure S4**. ROC curve in 3, 5 and 7 years. (A-C) All set (3 year, AUC = 0.754; 5 year, AUC = 0.707; 7 year, AUC = 0.797). (D-E) Training set (3 year, AUC = 0.874; 5 year, AUC = 0.786; 7 year, AUC = 0.786). (F-H) test set (3 year, AUC = 0.753; 5 year, AUC = 0.682; 7 year, AUC = 0.737).

**Figure S5**. Gene set enrichment analysis (GSEA) of ferroptosis-related lncRNA signature and enrichment analysis mechanism. (A-B) Regulatory mechanism of ferroptosis-related genes in cells. (C-G) Enriched pathways in the high-risk groups.

**Figure S6. Estimate score and distribution of immune cells**. (A) Estimate score of TCGA-STAD sample. The results showed ESTIMATE score was negatively correlated with tumor purity. (B) Scoring of different immune cells and immune-related pathways in high and low-risk groups. (C) Heatmap demonstrated the expression differences of various immune cells in each sample of TCGA-STAD. (D) Expression of m6A-related genes in high and low-risk groups. It was noteworthy that the expression of FTO (*p<*0.01), IGFBP3 (*p<*0.05), VIRMA (*p<*0.05) in high-risk group was higher than that in low-risk group. (E) Proportion of 22 immune cells in patient with gastric adenocarcinoma. The proportion of 22 immune cells in gastric adenocarcinoma samples was manifested by heatmap and box plot based on CIBERSORT algorithm. ^*^*p <* 0.05; ^**^*p <* 0.01; ^***^*p <* 0.001.

**Figure S7**. **The FPI score in low and high-risk groups, and the microsatellite instability in TCGA-STAD based on distribution of high and low FPI score.** (A) The survival rate of different FPI score based on high and low-risk groups in TCGA-STAD (n=198). (B) The survival rate between high-risk and low-risk groups with FPI>0 (n=122). (C) The survival rate between high-risk and low-risk groups with FPI<0 (n=76). (D-E) The distribution of different FPI score in microsatellite instability. The results manifested the proportion of MSI-H in the high-risk group (17%) was higher than that in the low-risk group (15%) in the FPI score group. (F) FPI score between gastric adenocarcinoma and normal tissue in TCGA-STAD (*p =* 8.3e-17).

**Figure S8**. **Mutation of ferroptosis-related genes in various pathways in gastric adenocarcinoma.** (A) High mutation related pathway. The results showed RTK-RAS, Hippo and TP53 pathways were vulnerable to STAD gene mutations. (B) PTK-RAS pathway. ERBB4 showed a high mutation state in RTK-RAS pathway. **(C)** NRF2 pathway. CUL3 was the highest mutation rate gene on NRF2 pathway. (D) TP53 pathway. The plot showed the high mutation rate of TP53, the mutation rate of ATM was higher in the TP53 pathway. (E) NOTCH pathway. The plot showed the mutation rate of CNTN6 was the highest. (F) PI3K pathway. The plot shown PIK3CA was the highest mutation rate gene. (G) Hippo pathway. both FAT3 and FAT4 showed high mutation status. (H) Cell Cycle pathway. The highest mutation rate gene was CDKN2A, it was consistent with our previous research conclusions. (I-J) TGF-Beta and WNT pathway. ACVR2A and APC showed high mutation in TGF-Beta pathway and WNT pathway, respectively.

**Figure S1**

**
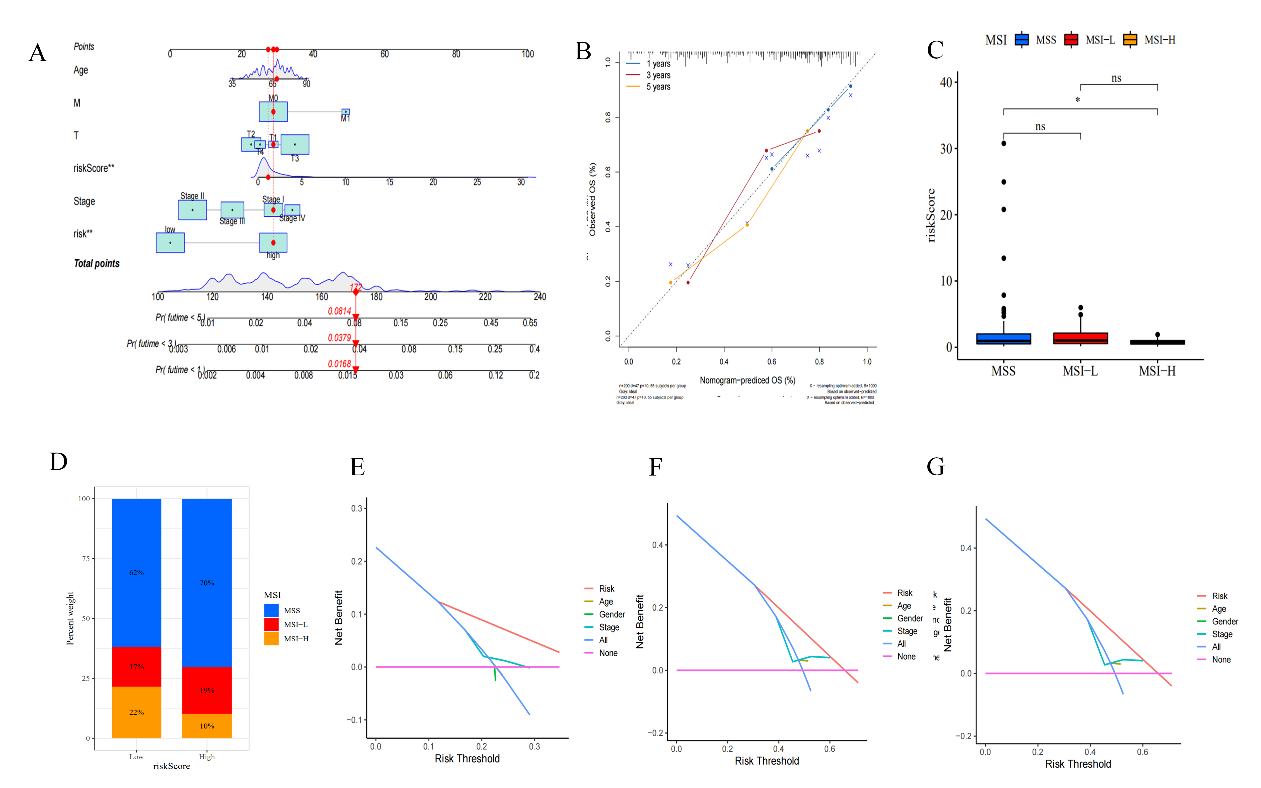
**

**Figure S2**

**
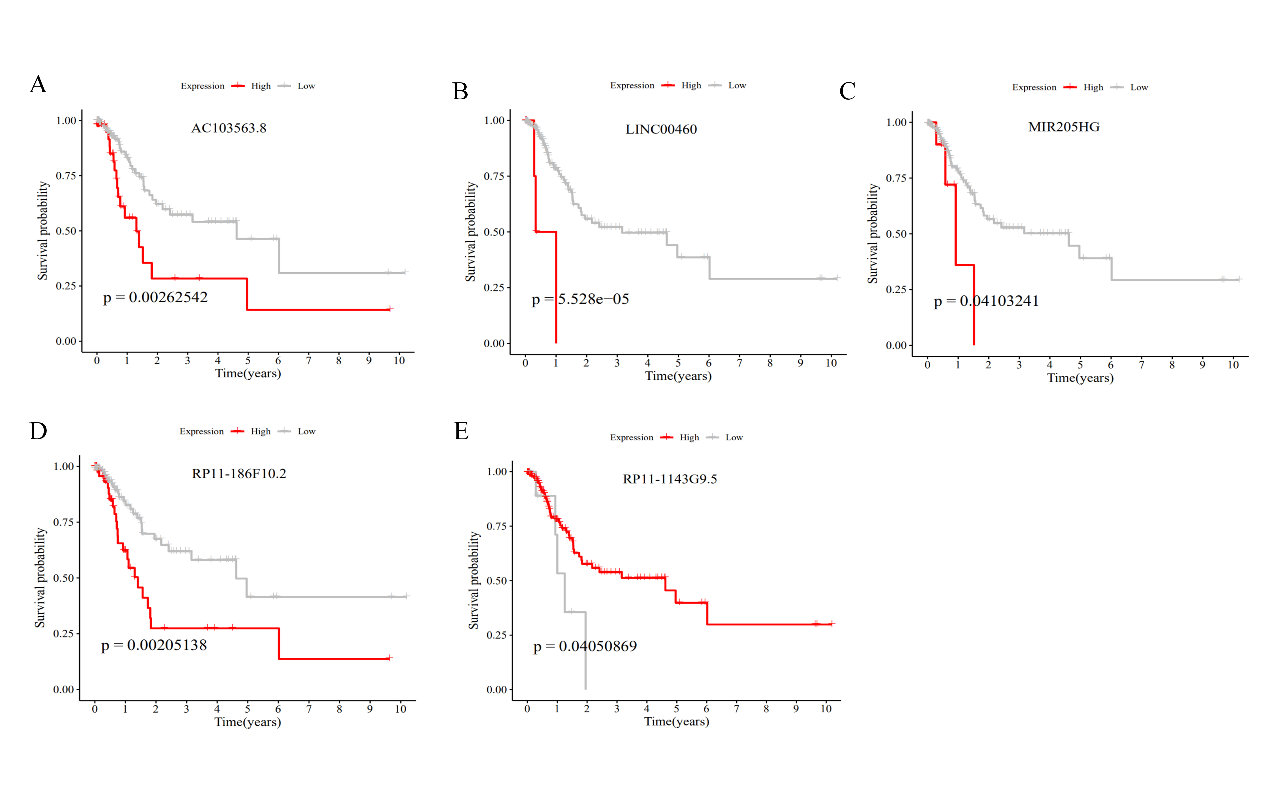
**

**Figure S3**

**
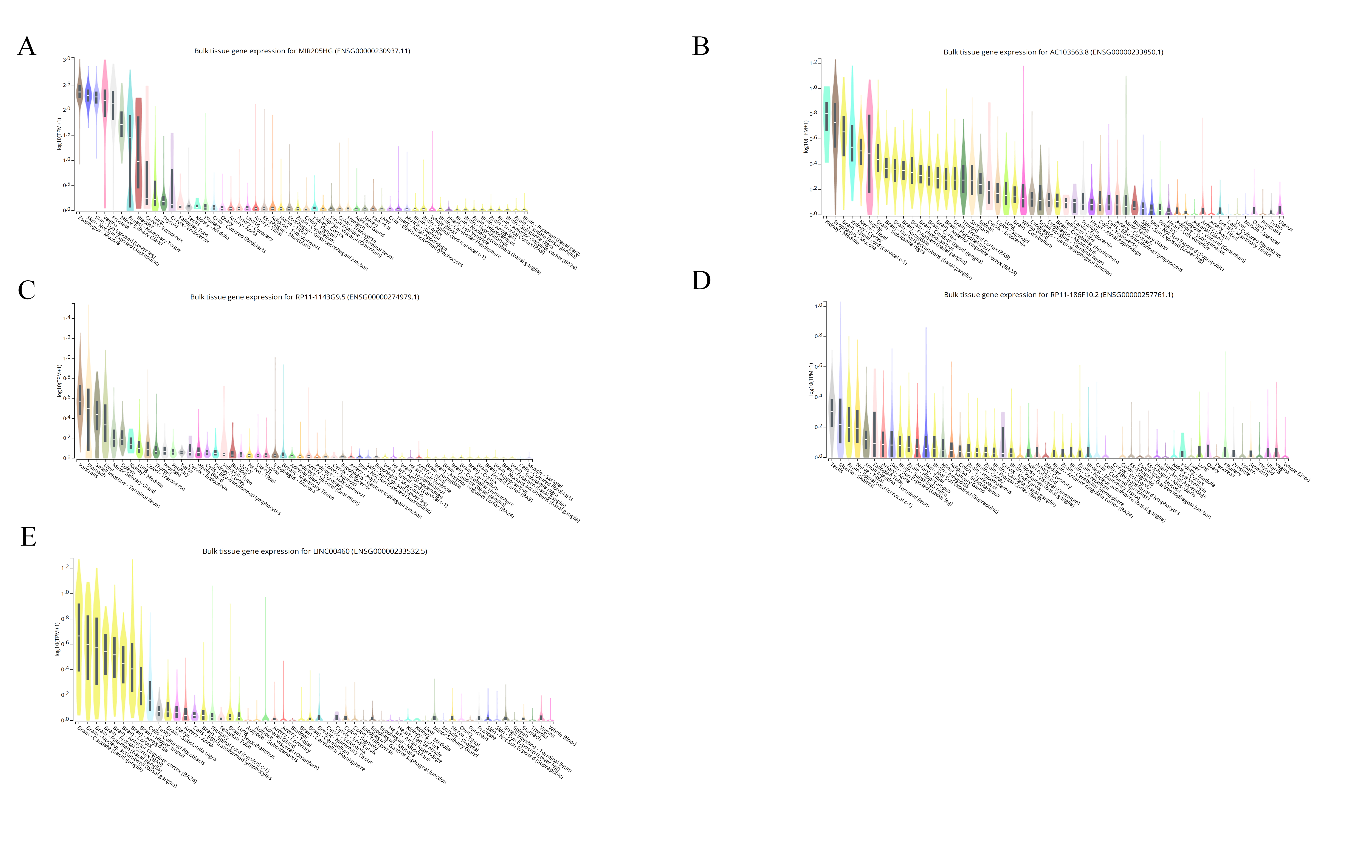
**

**Figure S4**

**
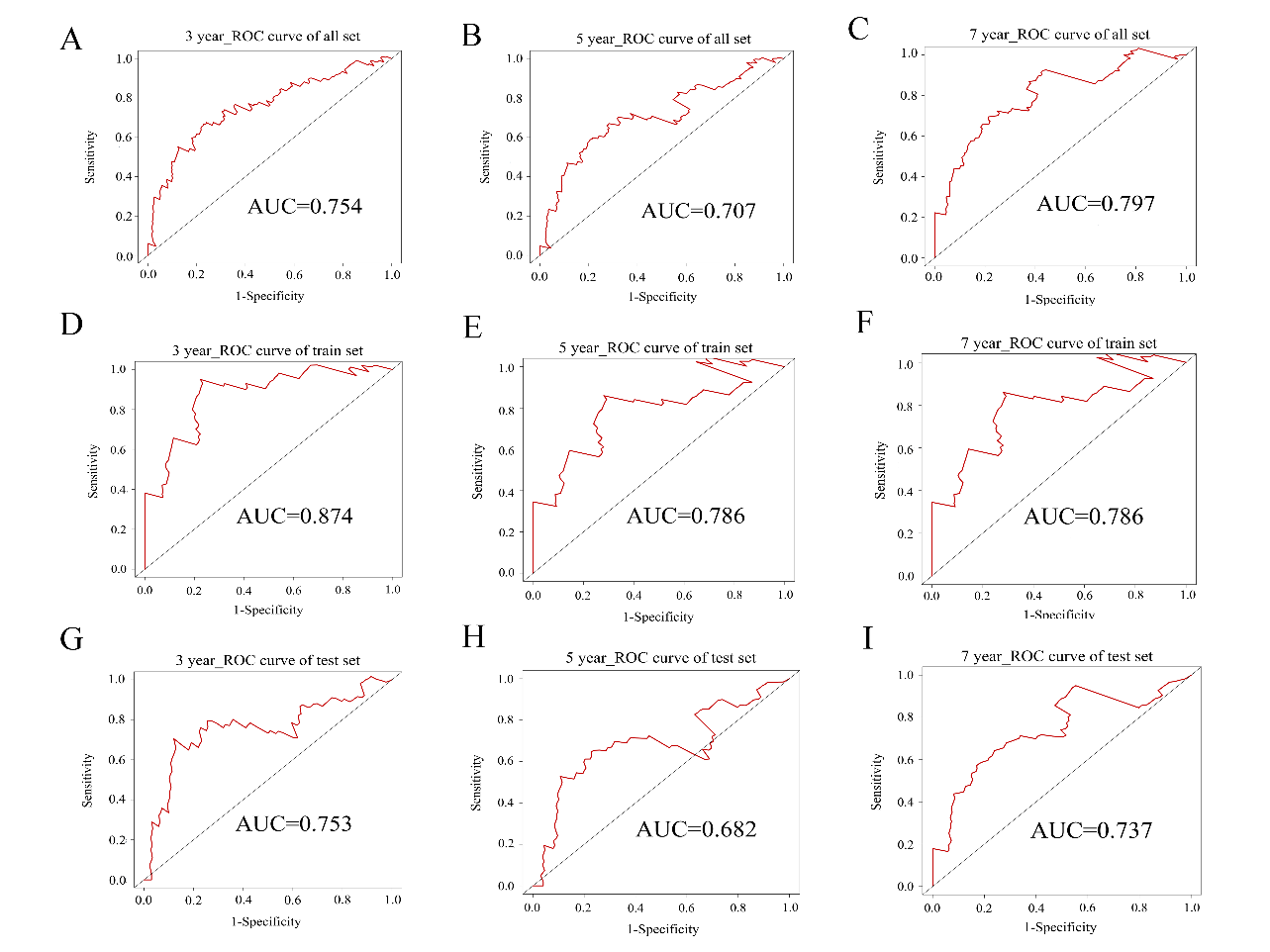
**

**Figure S5**

**
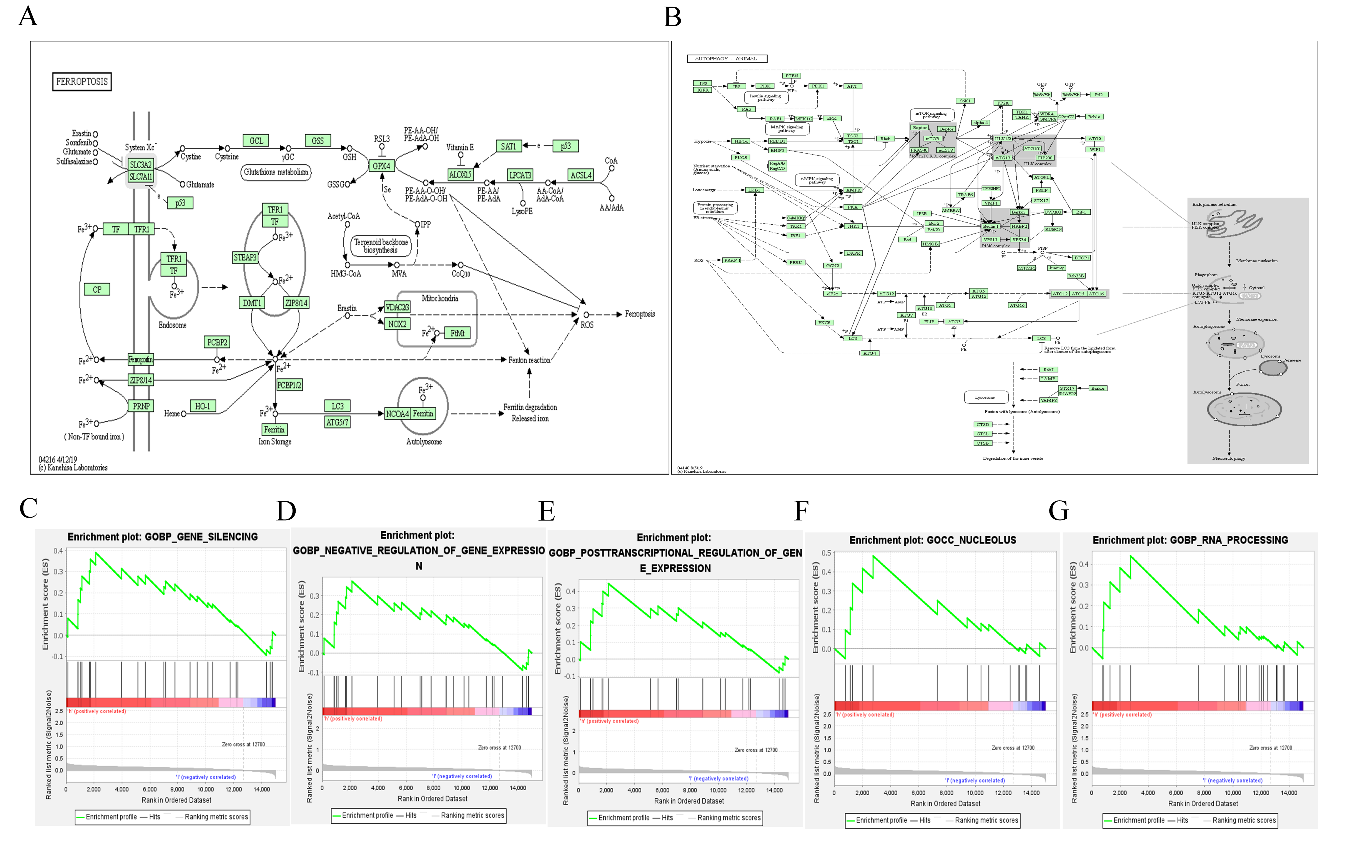
**

**Figure S6**

**
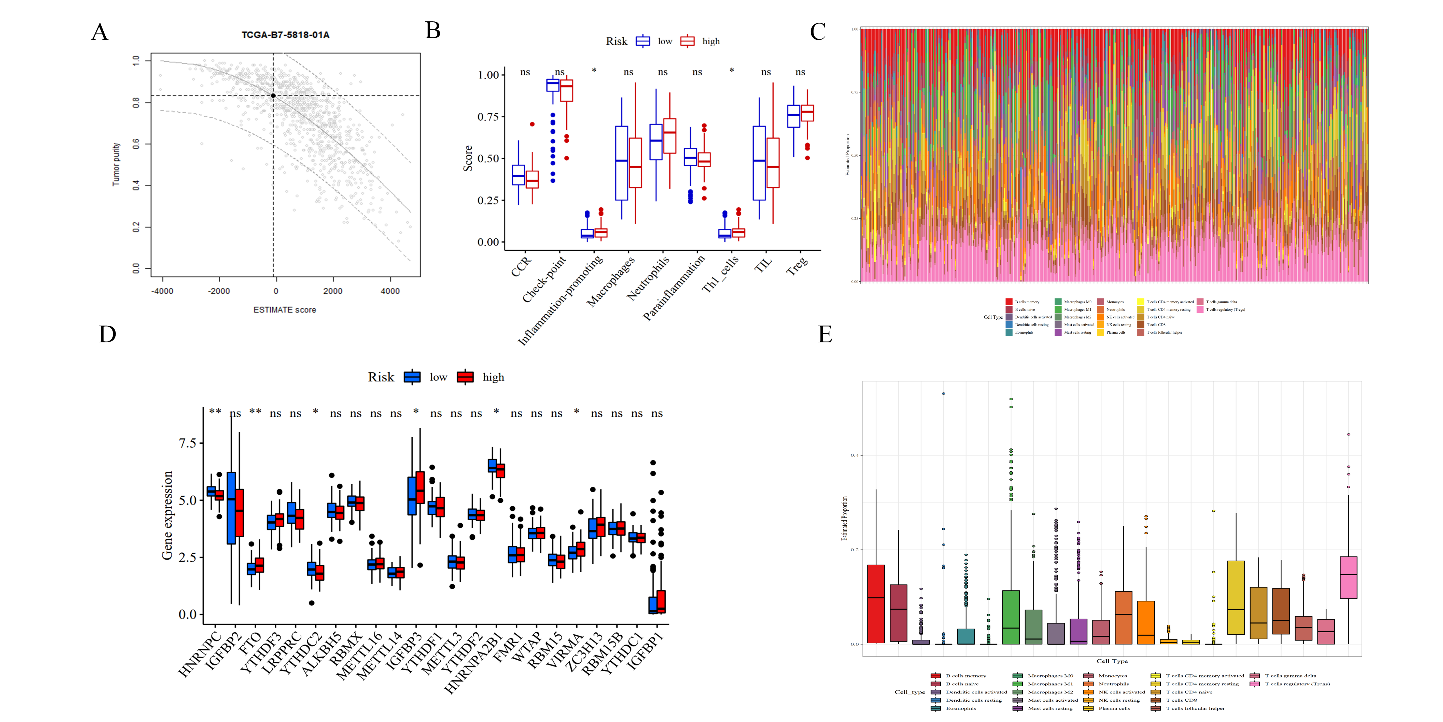
**

**Figure S7**

**
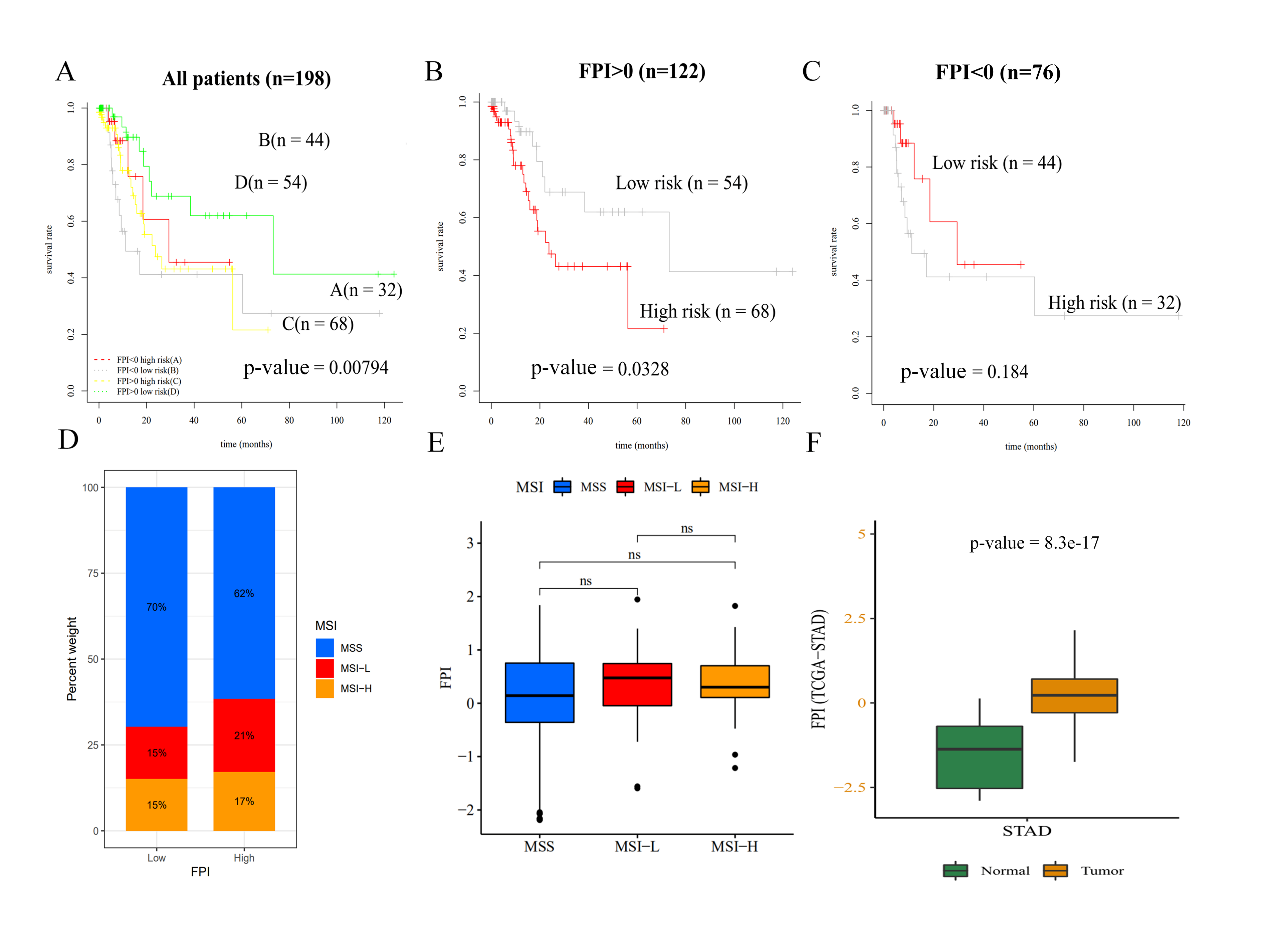
**

**Figure S8**

**
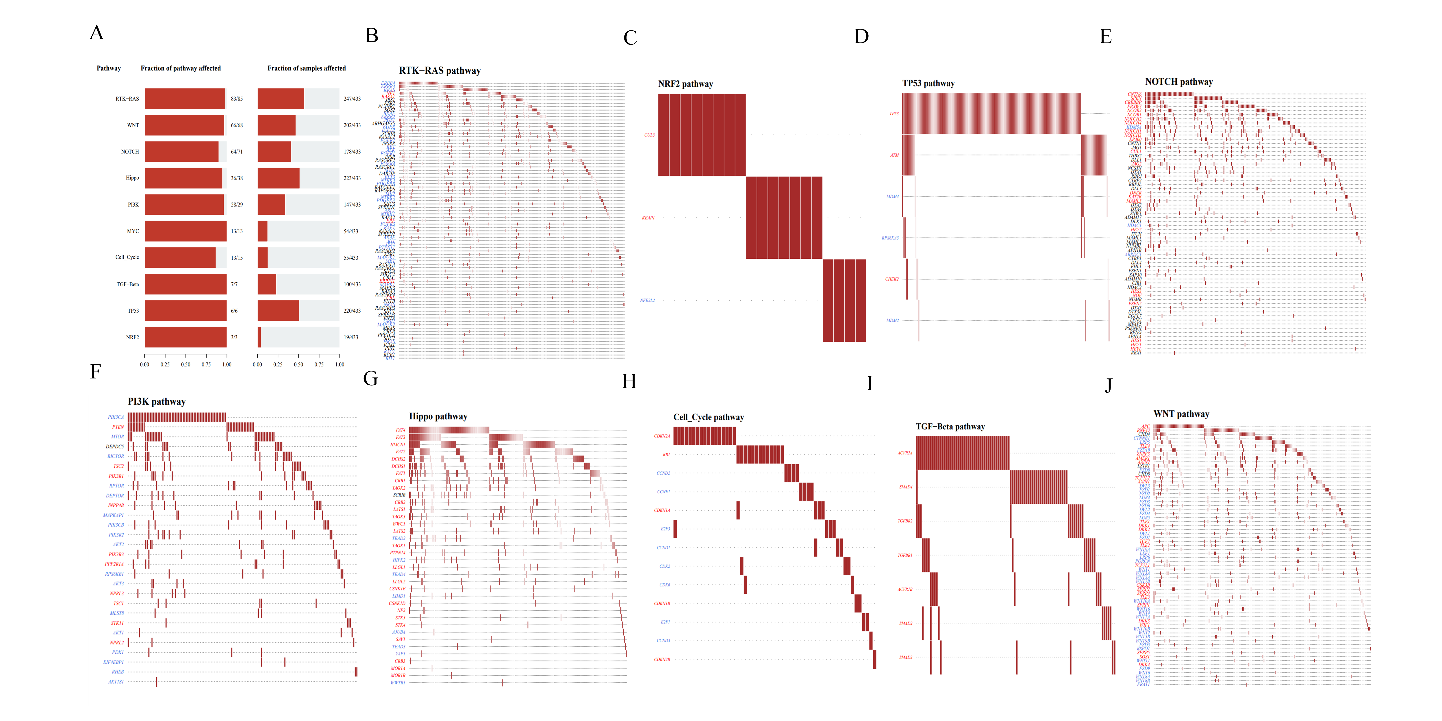
**

**Supplementary Tables**

**Supplementary Table S1**. Screening of 12 ferroptosis-related lncRNAs by Univariate Cox regression analysis.

| ***Gene*** | ***Coeffcient*** | ***HR*** | ***HR.95L*** | ***HR.95H*** | ***P-value*** |
| --- | --- | --- | --- | --- | --- |
| RP11-186F10.2 | 0.673325855337181 | 1.9607476514671 | 1.20885039010628 | 3.18032023168371 | 0.00636075663607458 |
| RP4-781K5.5 | 0.963959590558005 | 2.62205822272408 | 1.28111609947724 | 5.36656227032066 | 0.00834251626003355 |
| LINC01537 | 2.4422636077011 | 11.4990406218367 | 1.85723257596897 | 71.1962179285291 | 0.0086521824709508 |
| LINC00601 | 1.66379227662209 | 5.27929347188794 | 1.50296145625861 | 18.5440148489895 | 0.00944306469543467 |
| AC103563.8 | 0.605908029355259 | 1.83291579482645 | 1.13069181302806 | 2.97126084421459 | 0.0139591370409964 |
| AC103563.9 | 0.398962089208325 | 1.49027711995102 | 1.07028029781338 | 2.07508808560425 | 0.0181720114952298 |
| RP11-1143G9.5 | -0.376112894754809 | 0.686524823615547 | 0.501747962536684 | 0.93934877394923 | 0.0187190689225697 |
| LINC00460 | 0.302970714277203 | 1.35387481465918 | 1.03457948266221 | 1.77171212505758 | 0.027266720850285 |
| RP11-64B16.4 | 1.33845431518813 | 3.81314502759235 | 1.09968999809451 | 13.2219762175218 | 0.0348796000287955 |
| LINC00454 | 0.992809688119213 | 2.69880663459795 | 1.05194407598126 | 6.92390158113283 | 0.0388938127821046 |
| KB-68A7.1 | 0.745403470943598 | 2.10729149436973 | 1.03234207056844 | 4.30155620781571 | 0.0406196804555191 |
| MIR205HG | 0.272993862667218 | 1.31389218100644 | 1.01114619903695 | 1.70728294776171 | 0.041060985226122 |

**Supplementary Table S2.** Constructing the gastric adenocarcinoma prognosis signature based on 5 ferroptosis-related lncRNA by multivariate Cox regression analysis.

| **Gene** | **Coeffcient** | **HR** | **HR.95L** | **HR.95H** | ***P-value*** |
| --- | --- | --- | --- | --- | --- |
| RP11-1143G9.5 | -0.423917081201679 | 0.654478148200556 | 0.471034079392957 | 0.909364449859026 | 0.0115328319700428 |
| AC103563.8 | 1.2071928010986 | 3.34408395510372 | 1.34397485451501 | 8.32076393484133 | 0.00944241174144701 |
| LINC00460 | 0.40956647259787 | 1.50616467989272 | 1.07611452190751 | 2.10807678622827 | 0.0169585778548682 |
| RP11-186F10.2 | 1.07891999972853 | 2.94150101307087 | 1.71711942664938 | 5.03892045923706 | 0.0000854487546143532 |
| MIR205HG | -0.366418490206812 | 0.693212637798561 | 0.431324215749046 | 1.11411264115815 | 0.130127829645529 |

**Supplementary Table S3.** 12 drugs of TCGA-STAD were selected in GDSC, all of them p-value<0.05.

| ***Drug*** | ***p-value*** |
| --- | --- |
| JNJ.26854165 | 0.0037769636276552 |
| Imatinib | 0.00425877695672313 |
| CCT007093 | 0.00573258996514813 |
| KIN001.135 | 0.00618676112325527 |
| AP.24534 | 0.00635965169014988 |
| AMG.706 | 0.00692031807632788 |
| Lenalidomide | 0.00752232304293512 |
| Nilotinib | 0.0106294459433755 |
| JNK.Inhibitor.VIII | 0.01320046335195 |
| ABT.263 | 0.0260890860637317 |
| DMOG | 0.026536332096262 |
| AKT.inhibitor.VIII | 0.0372300441658142 |
